# Ameliorating Mitochondrial Dysfunction of Neurons by Biomimetic Targeting Nanoparticles Mediated Mitochondrial Biogenesis to Boost the Therapy of Parkinson's Disease

**DOI:** 10.1002/advs.202300758

**Published:** 2023-05-18

**Authors:** Qing Zheng, Hanghang Liu, Hao Zhang, Yaobao Han, Jiaxin Yuan, Tingting Wang, Yifan Gao, Zhen Li

**Affiliations:** ^1^ Center for Molecular Imaging and Nuclear Medicine State Key Laboratory of Radiation Medicine and Protection School for Radiological and Interdisciplinary Sciences (RAD‐X) Suzhou Medical College of Soochow University Collaborative Innovation Center of Radiation Medicine of Jiangsu Higher Education Institutions Suzhou 215123 China

**Keywords:** biomimetic nanoparticles, mitochondrial biogenesis, mitochondrial dysfunction, Parkinson's disease, SIRT1/PGC‐1*α* pathway

## Abstract

Mitochondrial dysfunction of neurons is the core pathogenesis of incurable Parkinson's disease (PD). It is crucial to ameliorate the mitochondrial dysfunction of neurons for boosting the therapy of PD. Herein, the remarkable promotion of mitochondrial biogenesis to ameliorate mitochondrial dysfunction of neurons and improve the treatment of PD by using mitochondria‐targeted biomimetic nanoparticles, which are Cu_2‐_ *_x_* Se‐based nanoparticles functionalized with curcumin and wrapped with DSPE‐PEG_2000_‐TPP‐modified macrophage membrane (denoted as CSCCT NPs), is reported. These nanoparticles can efficiently target mitochondria of damaged neurons in an inflammatory environment, and mediate the signaling pathway of NAD^+^/SIRT1/PGC‐1*α*/PPAR*γ*/NRF1/TFAM to alleviate 1‐methyl‐4‐phenylpyridinium (MPP^+^)‐induced neuronal toxicity. They can reduce the mitochondrial reactive oxygen species, restore mitochondrial membrane potential (MMP), protect the integrity of mitochondrial respiratory chain, and ameliorate mitochondrial dysfunction via promoting mitochondrial biogenesis, which synergistically improve the motor disorders and anxiety behavior of 1‐methyl‐4‐phenyl‐1,2,3,6‐tetrahydropyridine (MPTP)‐induced PD mice. This study demonstrates that targeting mitochondrial biogenesis to ameliorate mitochondrial dysfunction has a great potential in the treatment of PD and mitochondria‐related diseases.

## Graphical abstract

The neuronal mitochondrial dysfunction is the core pathogenesis of incurable Parkinson's disease (PD), and it is vital to ameliorate the mitochondrial dysfunction for boosting the therapy of PD. This work reports the amelioration of mitochondrial dysfunction and alleviation of PD symptoms by promoting neuronal mitochondrial biogenesis via NAD^+^/SIRT1/PGC‐1*α*/PPAR*γ*/NRF1/TFAM pathway through rationally designed mitochondria‐targeted biomimetic nanoparticles.

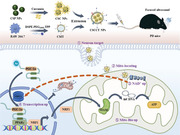

## 1 Introduction

Parkinson's disease (PD) is the second largest neurodegenerative disease with the features of loss of dopaminergic neurons in substantia nigra and striatum, cognitive decline and behavioral deficit.^[^ ^1^ ^]^ The occurrence and progression of PD involves various pathogenic mechanisms, including neuroinflammation, oxidative stress, *α*‐synuclein (*α*‐syn) aggregation, mitochondrial dysfunction, and impaired calcium homeostasis,^[^ ^2^ ^]^ of which the mitochondrial dysfunction is the core pathogenesis because mitochondria is the power house of neurons for their all activities.

Mitochondrial dysfunction can be caused by excessive reactive oxygen species (ROS), mitophagy defects, and the damage of mitochondrial transport, especially mitochondrial respiratory chain dysfunction.^[^ ^3^ ^]^ Mitochondrial respiratory chain is the electron transport chain (ETC) of mitochondrial oxidative phosphorylation, which is the center of mitochondria energy metabolism.^[^ ^4^ ^]^ Mitochondrial respiratory chain is located at the inner membrane of mitochondria and consists of five complexes, of which mitochondrial respiratory chain complex I (Complex I), also known as nicotinamide adenine dinucleotide dehydrogenase (NADH dehydrogenase), is the largest protein complex.^[^ ^5^, ^6^ ^]^ Recent studies have demonstrated that genetic knockout of Complex I can induce the mitochondrial dysfunction of neurons to initiate PD.^[^ ^7^ ^]^ In addition, damaging the Complex I by neurotoxins, such as 1‐methyl‐4‐phenyl‐1,2,3,6‐tetrahydropyridine (MPTP),^[^ ^8^ ^]^ rotenone,^[^ ^9^ ^]^ and 6‐hydroxydopamine (6‐OHDA),^[^ ^10^ ^]^ can also cause mitochondrial dysfunction, leading to mitophagy defects, oxidative stress, *α*‐syn aggregation, and other neuronal dysfunctions.^[^ ^11^ ^]^ Therefore, ameliorating mitochondrial dysfunction has emerged as an extremely promising approach for the treatment of PD, Alzheimer's disease (AD), and other mitochondria‐related diseases.^[^ ^12^, ^13^, ^14^, ^15^, ^16^ ^]^

One major strategy of ameliorating mitochondrial dysfunction is to eliminate impaired mitochondria by enhanced mitophagy. For example, Qin et al. used a novel peptide (Q14) to increase the neuronal mitophagy for treatment of PD.^[^ ^17^ ^]^ Xie et al. identified two autophagy inducers to enhance mitophagy for eliminating accumulated Amyloid‐*β* (A*β*) and neurofibrillary tangles in the brain.^[^ ^18^ ^]^ In addition to improving the mitophagy, another important strategy of ameliorating mitochondrial dysfunction is anti‐inflammation and antioxidation stress, because mitochondrial dysfunction can result in excessive ROS and cause serious inflammation and oxidative stress. In this context, some anti‐inflammatory and antioxidative agents have been extensively investigated. For example, chiral porous Cu*_x_* O nanoparticles exhibited excellent antioxidative capability of eliminating excessive ROS in MPTP‐induced PD mice.^[^ ^19^ ^]^ Superoxide dismutase (SOD) and catalase (CAT) based nanoenzymes could also effectively reduce cell apoptosis and mitochondrial dysfunction.^[^ ^20^ ^]^

Recent studies on Parkin‐deficient human dopaminergic neurons showed that mitochondrial dysfunction is the result of defective mitochondrial biogenesis.^[^ ^21^, ^22^ ^]^ Mitochondrial biogenesis controls the quality of mitochondria, and involves the synthesis of encoded proteins in the inner and outer membranes of mitochondria, nuclear‐encoded proteins, and the replication of mitochondrial DNA (mtDNA).^[^ ^23^ ^]^ It is the key biological process to maintain the number and function of mitochondria that play a crucial role in cell growth.^[^ ^24^, ^25^ ^]^ Further research demonstrates that the upregulation of Parkin interacting substrate and downregulation of peroxisome proliferator‐activated receptor‐*γ* coactivator‐1*α* (PGC‐1*α*) are the main causes of defective mitochondrial biogenesis.^[^ ^26^ ^]^ Additionally, AMPK, SIRT1, Cdc4, and other proteins can activate the nuclear regulation of PGC‐1*α* after phosphorylation, deacetylation, and ubiquitination, respectively. Among them, SIRT1 is the only protein that can sense the change of oxidized nicotinamide adenine dinucleotide (NAD^+^),^[^ ^27^ ^]^ and mediate the communication and cooperation between mitochondria and nucleus accordingly, which means that SIRT1/PGC‐1*α* is very important for mitochondrial biogenesis. Therefore, improving mitochondrial biogenesis via PGC‐1*α* is expected to solve the issue of mitochondrial dysfunction from the root, and significantly improve the efficacy of PD treatment.

Despite of the promise of improving mitochondrial biogenesis in treatment of PD, there are few studies on this theme because of difficulties in accurately regulating the pathway of mitochondrial biogenesis and the unclear molecular mechanism. More importantly, the currently available therapeutic agents cannot efficiently accumulate in the lesion area of substantia nigra and striatum, and cannot target the mitochondria of damaged neurons. Both the small molecule and nanoscale therapeutic agents such as polyphenols,^[^ ^28^ ^]^ melatonin^[^ ^29^ ^]^ derives, and iron oxide (Fe_3_O_4_) nanoparticles^[^ ^30^ ^]^ face the problems of low targeting capability and less efficacy. For example, curcumin is a natural polyphenol explored for the treatment of neurodegenerative disease,^[^ ^31^, ^32^, ^33^ ^]^ and inflammation diseases.^[^ ^34^, ^35^, ^36^ ^]^ However, its poor targeting capability, poor water solubility, low bioavailability, and short pharmacokinetic dynamics limit its in vivo therapeutic efficacy and clinical application.^[^ ^37^ ^]^ Therefore, there is an urgency to develop novel agents with excellent mitochondria‐targeted capability to solve mitochondrial dysfunction of neurons for boosting the treatment of PD.

**Scheme 1:**
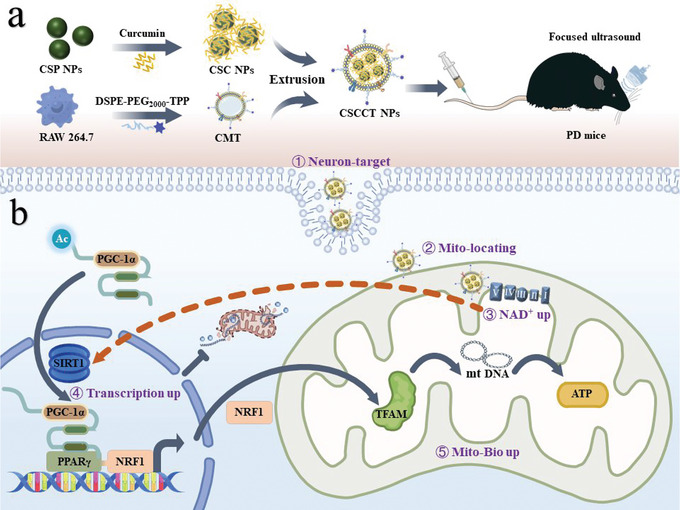
Schematic illustration of ameliorating mitochondrial dysfunction of neurons by biomimetic targeting nanoparticles mediated mitochondrial biogenesis to boost therapy of Parkinson's disease. a) Synthesis of biomimetic targeting nanoparticles (CSCCT NPs). b) CSCCT NPs protect against mitochondrial dysfunction by NAD^+^/SIRT1/PGC‐1*α*/PPAR*γ*/NRF1/TFAM pathway.

In our previous reports, quercetin‐functionalized Cu_2‐_ *_x_* Se nanoparticles have been successfully used to eliminate ROS and polarize the proinflammatory M1‐type microglia into anti‐inflammatory M2‐type ones for treatment of PD.^[^ ^38^ ^]^ Additionally, the excellent photothermal conversion performance of Cu_2‐_ *_x_* Se nanoparticles was used to controllably open the surface TRPV1 channels on microglia to enhance their autophagy for effective clearance of *α*‐syn aggregates.^[^ ^39^ ^]^ In this paper, we report the amelioration of mitochondrial dysfunction of neurons to boost PD therapy by improving mitochondrial biogenesis through rationally designed mitochondria‐targeted nanoparticles (**Scheme** **1**). The designed mitochondria‐targeted nanoparticles are based on ultrasmall Cu_2‐_ *_x_* Se nanoparticles,^[^ ^40^ ^]^ which can not only serve as copper‐ and selenium sources for synthesis of endogenous antioxidants (e.g., Cu/Zn SOD and glutathione peroxidase 4 (Gpx4),^[^ ^41^, ^42^ ^]^ but also efficiently degrade H_2_O_2_ into H_2_O through their Fenton‐like reaction.^[^ ^43^, ^44^ ^]^ In addition, the ultrasmall Cu_2‐_ *_x_* Se nanoparticles can efficiently carry curcumin through the strong coordination between curcumin and surface copper cations to overcome the aforementioned curcumin shortcomings.^[^ ^45^, ^46^ ^]^ The curcumin modified Cu_2‐_ *_x_* Se nanoparticles (denoted as CSC NPs) with NADH oxidase (NOX)‐like activity were wrapped by DSPE‐PEG_2000_‐TPP‐modified macrophage membranes to endow them with capabilities of targeting inflammatory neurons and their mitochondria. The resultant nanoparticles (abbreviated as CSCCT NPs) can be efficiently delivered to the inflammatory site with the assistance of focused ultrasound (US), and then specifically targeted the mitochondria of inflammatory neurons to improve their mitochondrial biogenesis. They can improve the ratio of NAD^+^ to NADH in the mitochondria of neurons, and increase the expression of NAD^+^‐dependent deacetylase sirtuin‐1 (SIRT1) in the nuclei, thereby regulating the gene expression of mitochondrial biogenesis at the transcriptional level to improve PD therapy. Our study demonstrates that targeted enhancement of mitochondrial biogenesis is a promising approach for treatment of PD and other mitochondria‐related diseases.

## 2 Results and Discussion

### 2.1 Synthesis, Characterization, and Cytotoxicity of CSC NPs

The ultrasmall Cu_2‐_ *_x_* Se nanoparticles modified by polyvinylpyrrolidone (abbreviated as CSP NPs) were synthesized in an aqueous solution under ambient conditions and nitrogen protection as reported elsewhere.^[^ ^40^ ^]^ CSP NPs were characterized by transmission electron microscope (TEM) to show a uniform size (2.1 ± 0.3 nm, Figure S1a,b, Supporting Information). They were characterized by powder X‐ray diffraction (XRD) to be cubic berzelianite with weak diffraction peaks (Cu_2‐_ *_x_* Se, JCPDS Card No. 06–0680, Figure S1c, Supporting Information). Their crystal structure was further determined by high‐resolution transmission electron microscope (HRTEM) to show a lattice spacing of 0.2 nm, which is matched well with that of (220) planes of cubic berzelianite (Cu_2‐_ *_x_* Se) (Figure S1d, Supporting Information). Additionally, energy dispersive spectrometer (EDS) was applied for determining the atomic ratio of Cu to Se, which is 1.8 (*x* = 0.2) and means the existence of copper defects (Figure S1e, Supporting Information). These results indicate the successful synthesis of CSP NPs.

**Figure 1:**
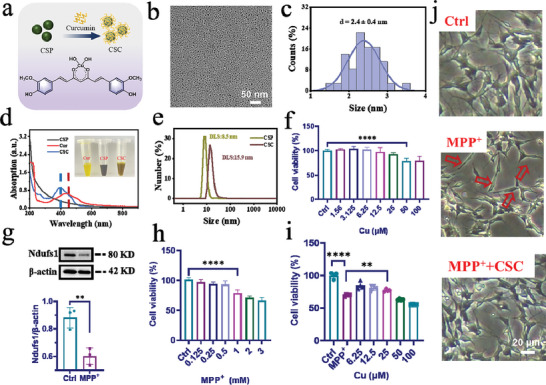
Synthesis, characterization, and cytotoxicity of CSC NPs. a) Schematic illustration of the preparation of CSC NPs and the chelation between Cu^2+^ and Cur. b,c) TEM image and particle size distribution of CSC NPs. d) UV–vis absorption of Cur, CSP NPs, and CSC NPs and solutions with inset of their corresponding digital optical images. e) Hydrodynamic size of CSP NPs and CSC NPs. f) Cell viability of SH‐SY5Y cells after treatment with different concentrations of CSC NPs (*n* = 4). g) Western blot and quantitative analysis of Ndufs1 protein in SH‐SY5Y cells incubated with 3 mm MPP^+^ for 24 h (*n* = 3). h) Cell viability of SH‐SY5Y cells after treatment with different concentrations of MPP^+^ (*n* = 4). i) Cell viability of SH‐SY5Y cells after pretreatment with different concentrations of CSC NPs, and then treatment with 3 mm MPP^+^ (*n* = 4). j) Representative morphology images of the SH‐SY5Y cells, the cells incubated with MPP^+^, and the cells pretreated with CSC NPs and then incubated with MPP^+^, which was pictured by an inverted microscope. The two‐sided unpaired Student's *t*‐test was used for comparison between two groups. The two‐sided one‐way ANOVA with a Tukey post hoc analysis was used for comparison among multiple groups. *P < 0.05, **P < 0.01, ***P < 0.001, ****P < 0.0001, ns, not significant.

The ultrasmall CSP NPs have a large surface area and a plenty of surface Cu^2+^ for chelating with curcumin (Cur) to form CSP‐Cur nanoparticles (abbreviated as CSC NPs) (**Figure** **1**a). The size of CSC NPs was measured to be (2.4 ± 0.4) nm by TEM (Figure 1b,c), which indicates their excellent dispersibility and homogeneity. The hydrodynamic size of nanoparticles determined by dynamic light scattering (DLS) was increased from 8.5 to 15.9 nm after modification with Cur (Figure 1e), accompanied with the change of zeta potential from −15 to −23 mV (Figure S2a, Supporting Information). Moreover, the characteristic ultraviolet–visible (UV–vis) absorption of Cur was blue shifted from 436 to 396 nm (Figure 1d), due to the chelation of Cur with surface Cu^2+^ on nanoparticles, which decreased its conjugation degree and increased the steric hindrance. The notable color difference in the solids and solutions of Cur, CSP NPs, and CSC NPs (Figure S2b, Supporting Information; inset in Figure 1d) also demonstrates the successful modification of CSP NPs with Cur. Their Fourier transform infrared spectroscopy (FTIR) spectra also verify the successful surface modification with Cur. The spectrum of CSP NPs clearly shows the polymer characteristic vibration of C—H bonds at ≈2800 cm^−1^ and the characteristic vibration peak of C=O bonds at ≈1650 cm^−1^, while the spectrum of pure Cur shows obvious characteristic vibration of benzene skeleton below 1000 cm^−1^ and that of C=O and C=C bonds at ≈1650 cm^−1^ and ≈1500 cm^−1^. All above characteristic vibrations were observed in the spectrum of CSC NPs, which indicates the successful synthesis of CSC NPs (Figure S3a, Supporting Information). The content of Cur in CSC NPs was estimated to be 16.5 wt% by thermosgravimetric analysis (TGA, Figure S3b, Supporting Information).

The above results collectively prove the successful modification of CSP NPs with Cur. The total antioxidative capability of CSC NPs was evaluated by using 2,2′‐azino‐bis (3‐ethylbenzthiazoline‐6‐sulfonic acid (ABTS), which can be oxidized into green ABTS^+^ by the ROS. Their antioxidative performance was elevated gradually with the increase of CSC NPs concentration (Figure S2c, Supporting Information), indicating that CSC NPs may protect mitochondria from damage. We also detected the enzyme‐like activity of CSC nanoparticles such as SOD activity for clearing hydroxyl radical, or NOX activity for catalyzing the oxidation of NADH intoNAD^+^. The results show that Cur, CSP NPs, and CSC NPs all had weak SOD‐like activity (Figure S4a, Supporting Information), while CSC NPs had strong NOX‐like activity mainly contributed by CSP NPs (Figure S4b, Supporting Information).

Based on above results, we assessed the cytotoxicity of CSC NPs to SH‐SY5Y cells. The survival ratio of SH‐SY5Y cells was decreased with the concentration of CSC NPs increasing from 1.56 to 100 µm (Figure 1f), which was calculated according to the linear relationship between their absorbance at 396 nm and the concentration (Figure S3c,d, Supporting Information). 25 µm CSC NPs were selected for the later experiments. The cellular model of neuron injury with the destroyed Complex I was established by culturing SH‐SY5Y cells with 1‐methyl‐4‐phenylpyridinium (MPP^+^), which can damage the mitochondria of dopaminergic neurons.^[^ ^47^ ^]^ To determine whether MPP^+^ damaged the mitochondrial Complex I, the expression of Ndufs1 (NADH: ubiquinone oxidoreductase core subunit S1) was analyzed by Western blot, which showed ≈1.5‐fold decrease (Figure 1g) and proved the mitochondrial damage in the cells. The toxicity of different concentrations of MPP^+^ to SH‐SY5Y cells was assessed by using CCK‐8 method. The cell viability was higher than 50% after incubation for 24 h when the concentration of MPP^+^ was lower than 3 mm (Figure 1h). Therefore, 3 mm MPP^+^ was selected for the subsequential cellular experiments.

To demonstrate the protective role of CSC NPs, we pretreated SH‐SY5Y cells with CSC NPs, and then cultured with 3 mm MPP^+^ for 24 h. The higher cell viability than that obtained without pretreatment with CSC NPs demonstrates the excellent protective effect of CSC NPs on cells (Figure 1i). In addition, the morphology of SH‐SY5Y cells cultured with MPP^+^ was changed from the stringy shape to the normal multiprotrusion type after they were pretreated with 25 µm CSC NPs for 2 h (Figure 1j), indicating that CSC NPs can protect them from damage caused by MPP^+^.

### 2.2 CSC NPs Protect Neuronal Mitochondria from Damage Caused by MPP^+^

**Figure 2:**
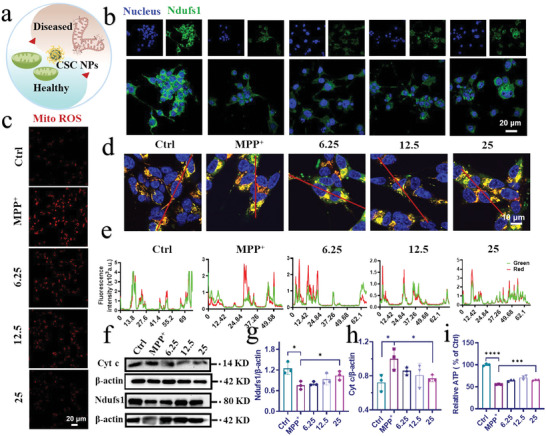
CSC NPs alleviated the cell apoptosis and mitochondrial damage caused by MPP^+^. a) Schematic illustration of MPP^+^‐induced diseased mitochondria recovering into healthy mitochondria by incubation with CSC NPS. b) Confocal laser scanning microscope (CLSM) images of the expression of Ndufs1 protein in SH‐SY5Y cells treated with different concentrations of CSC NPs and incubated with MPP^+^ for 24 h. Blue: cell nuclei; green: Ndufs1. c) CLSM images revealed the level of mitochondrial ROS generated in SH‐SY5Y cells pretreated with different concentrations of CSC NPs and incubated with MPP^+^ for 24 h. Red: Mito ROS. d,e) CLSM images and colocation analysis indicated total and active mitochondria of SH‐SY5Y cells pretreated with different concentrations of CSC NPs and then incubated with MPP^+^. Blue: cell nuclei; green: total mitochondria; red: active mitochondria. f) Western blot and g,h) quantification analysis of Cyt c and Ndufs1 in SH‐SY5Y cells pretreated with different concentrations of CSC NPs and then incubated with MPP^+^ (*n* = 3). i) Relative level of ATP in SH‐SY5Y cells pretreated with CSC NPs and then incubated with MPP^+^ (*n* = 3). The two‐sided one‐way ANOVA with a Tukey post hoc analysis was used for comparison among multiple groups. *P < 0.05, **P< 0.01, ***P < 0.001, ****P < 0.0001, ns, not significant.

Mitochondria play a crucial role in the brain energy metabolism as Ca^2+^ sinks and anabolic factories.^[^ ^48^ ^]^ On the basis of above results, we explored whether CSC NPs could prevent mitochondrial damage from multiple perspectives (**Figure** **2**a). First, the largest subunit of Complex I Ndufs1, which locates at the mitochondrial inner membrane and serves as an important component in the iron–sulfur fragment of the enzyme,^[^ ^49^ ^]^ was investigated by immunofluorescence assay (Figure 2b). The fluorescence intensity of Ndufs1 in the SH‐SY5Y cells treated with 3 mm MPP^+^ was dramatically decreased. However, it was gradually increased with the concentration of CSC NPs changing from 6.25 to 25 µm (Figure S5a, Supporting Information). These results are consistent with that of the Western blot (Figure 2f,g), and demonstrate that CSC NPs can attenuate mitochondrial damage caused by neurotoxin MPP^+^ in a concentration dependent manner.

It is well known that the destroy of mitochondrial respiratory chain can generate a large amounts of ROS (such as ∙OH and O_2_∙^−^, etc.) in mitochondria, resulting in redox imbalance and the death of cells.^[^ ^50^ ^]^ We tested the change of intracellular mitochondrial hydroxyl radical (∙OH) after pretreatment with CSC NPs, which is the most reactive ROS, by using MitoROS OH580 as a probe. The MitoROS OH580 can react with ∙OH to show strong red fluorescence. The fluorescence intensity of cells pretreated with 25 µm CSC NPs for 2 h and then incubated with 3 mm MPP^+^ for 24 h was comparable to that of normal cells without any treatment, which demonstrates that CSC NPs protected SH‐SY5Y cells by reducing the excess mitochondrial ROS caused by MPP^+^ (Figure 2c; Figure S5b, Supporting Information). In addition, mitochondria as an important organelle have many antioxidative enzymes to modulate ROS and keep redox balance. We tested the manganese superoxide dismutase superoxide dismutase 2 (SOD2) in mitochondria at genetic level to evaluate the function of mitochondria protected by CSC NPs, because SOD2 exists widely in mitochondria and protects mtDNA from oxidation. When SH‐SY5Y cells were pretreated with 25 µm CSC NPs for 2 h and then incubated with MPP^+^, the mRNA level of SOD2 was significantly recovered to that of normal cells, in comparison with MPP^+^‐treated cells, which showed a drastic decrease in SOD2 (Figure S6a, Supporting Information). These results indicate the enhanced protective effect of SOD2 on mitochondria and cells, and the mechanism needs to be further explored.

We further determined the difference and variation in the number and activity of mitochondria under different conditions with two kinds of probes. The green fluorescence and red fluorescence represent total and active mitochondria, respectively, and their merged orange fluorescence indicates the ratio of active mitochondria. As shown in Figure 2d, the normal SH‐SY5Y cells showed more orange fluorescence, indicating high ratio of active mitochondria. However, the MPP^+^‐treated cells showed low ratio of active mitochondria, evidenced by weak red fluorescence and strong green fluorescence. The fluorescence difference between treated and untreated cells was resulted from the destroy of mitochondria by MPP^+^. Interestingly, the fluorescence of cells pretreated with CSC NPs and then incubated with 3 mm MPP^+^ was close to that of untreated cells, and their similarity was increased with the concentration of CSC NPs. We further evaluated their colocalization by fluorescence quantification to show the improvement of mitochondrial function. After pretreatment of cells with 25 µm CSC NPs, the red fluorescence of active mitochondria was maximally overlapped with the green fluorescence of total mitochondria to result in strong orange fluorescence (Figure 2e). These results demonstrate that CSC NPs can effectively protect mitochondria from damage caused by MPP^+^.

To further assess the protective effect of CSC NPs on mitochondria, the expression of cytochrome c (Cyt c), which plays a crucial role in activation of apoptosis,^[^ ^51^ ^]^ was detected via Western blot. The Western blot and quantification results show that Cyt c in the cells treated with MPP^+^ for 24 h was higher than that of normal cells without treatment, which was reduced to the normal level when the cells were pretreated with various concentrations of CSC NPs (Figure 2f,h). The decreased Cyt c suggests the less occurrence of caspase‐3‐dependent apoptosis. Thus, immunofluorescence was used to test the activated and cleaved‐caspase3 (C‐caspase3). Caspase‐3 is a family of cysteine proteases that are the key mediators of programmed cell death or apoptosis.^[^ ^52^ ^]^ Compared with the cells incubated with MPP^+^ for 24 h alone, the fluorescence intensity of the cells pretreated with 25 µm CSC NPs for 2 h and then treated with 3 mm MPP^+^ was declined to the level of cells without any treatment (Figure S6b,c, Supporting Information). These results explain why CSC NPs can increase the survival of SH‐SY5Y cells exposed to MPP^+^ in the cytotoxicity assay.

Besides the amount of active mitochondria, the function of mitochondria was evaluated by their capability of synthesizing adenosine triphosphate (ATP), which is a gold standard for assessment. Figure 2i shows that 3 mm MPP^+^ was sufficient to destroy the mitochondrial respiratory chain of SH‐SY5Y cells and reduce their capability of producing ATP, but CSC NPs effectively ameliorated the mitochondrial function of cells and prevented them from damage by neurotoxin. The recovery of mitochondrial productivity indicates that mitochondria were well protected by CSC NPs in a concentration dependent manner.

### 2.3 CSC NPs Regulate SIRT1/PGC‐1*α* Pathway to Protect Mitochondria

**Figure 3:**
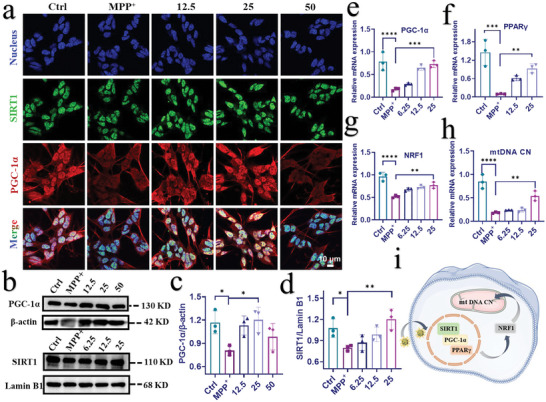
CSC NPs ameliorated MPP^+^ ‐induced mitochondrial dysfunction through the SIRT1/PGC‐1*α* pathway. a) CLSM images indicated the expression of SIRT1 and PGC‐1*α* proteins in SH‐SY5Y cells pretreated with different concentrations of CSC NPs and then incubated with MPP^+^. Blue: cell nuclei; green: SIRT1; red: PGC‐1*α*. b) Western blot and c,d) quantification of SIRT1 and PGC‐1*α* proteins in SH‐SY5Y cells pretreated with different concentrations of CSC NPs and incubated with MPP^+^ (*n* = 3). e–h) Gene expressions of PGC‐1*α*, PPAR*γ*, NRF1, and mitochondrial copy number (mtDNA CN) analyzed by qRT‐PCR (*n* = 3). i) Schematic illustration of the mechanism of CSC NPs improving mitochondrial dysfunction and biogenesis via SIRT1/PGC‐1*α* pathway. The two‐sided one‐way ANOVA with a Tukey post hoc analysis was used for comparison among multiple groups. *P < 0.05, **P < 0.01, ***P < 0.001, ****P < 0.0001, ns, not significant.

The previous results demonstrate that CSC NPs can well protect mitochondria from damage, and it is of great importance to investigate their protective mechanism. It is well known that mitochondrial biogenesis is mainly regulated by PGC‐1*α*, and improving mitochondrial biogenesis can protect cells from damage.^[^ ^53^ ^]^ Therefore, the PGC‐1*α* and its upstream related regulator sirtuin 1 (SIRT1), which is one of the mammalian homologues of yeast silent information regulator 2 (Sir2), and dependent on the NAD^+^,^[^ ^54^ ^]^ were determined to demonstrate the protection of CSC NPs on mitochondria by confocal laser scanning microscope (CLSM). The PGC‐1*α* can be deacetylated by SIRT1 under the assistance of coenzyme NAD^+^.^[^ ^54^ ^]^ We used different concentrations of CSC NPs (12.5, 25, 50 µm) to incubate with SH‐SY5Y cells for 2 h in advance, and then cultured the cells with 3 mm MPP^+^ for 24 h. Both PGC‐1*α* and SIRT1 were examined by immunofluorescence (**Figure** **3**a; Figure S7a, Supporting Information), which clearly shows that MPP^+^ treatment resulted in a decrease in the fluorescence intensity of SIRT1 and PGC‐1*α* compared with that of untreated cells. By contrast, CSC NPs promoted the expression of SIRT1, which was increased with the concentration from 12.5 to 25 µm. Further increasing the concentration of CSC NPs from 25 to 50 µm decreased the SIRT1 level due to the cytotoxicity of nanoparticles at high concentration. The Western blot of SIRT1 was consistent with immunofluorescence result (Figure 3b–d), which confirms that the optimal concentration of CSC NPs was 25 µm. After quantitatively analyzing the fluorescence intensity of PGC‐1*α* (Figure S7b, Supporting Information) and Western blot (Figure 3b,c), we found that the change of PGC‐1*α* was same as that of SIRT1. The amount of PGC‐1*α* translocating into the nucleus from the cytoplasm was also significantly reduced in the cells treated with MPP^+^ alone, which was recovered by pretreating cells with 25 µm CSC NPs. It should be noted that the translocation of PGC‐1*α* into the nucleus is deacetylated by SIRT1 to activate genetic transcription. Based on these results, we conclude that the mitochondrial protection by CSC NPs was related to PGC‐1*α*‐mediated mitochondrial biogenesis.

In the next, we used the quantitative real‐time polymerase chain reaction (qRT‐PCR) to characterize the change of PGC‐1*α* at genetic level (Figure 3e). Similar to the protein expression, the mRNA expression of PGC‐1*α* was drastically decreased in the cells treated with MPP^+^ in comparison with the normal cells. After pretreatment with CSC NPs, the mRNA expression of PGC‐1*α* in the cells was gradually increased with the concentration of CSC NPs and reached to the normal level. These results demonstrate that CSC NPs can modulate PGC‐1*α* at genetic level.

The upregulation of PGC‐1*α* can activate its downstream gene peroxisome proliferator activated receptor *γ* (PPAR*γ*) to regulate multiple transcriptional factors. To prove the influence of PGC‐1*α* on PPAR*γ*, we also detected the mRNA expression of PPAR*γ* via qRT‐PCR. Figure 3f shows that the mRNA level of PPAR*γ* in the normal cells was 12.7‐fold of that in the MPP^+^‐treated cells. After the cells were pretreated with CSC NPs, their mRNA level of PPAR*γ* was greatly improved, especially for the cells pretreated with 25 µm CSC NPs, which had a similar level to that of normal cells.

The above results demonstrate that PPAR*γ* was activated by PGC‐1*α*, which was translocated into nucleus and deacetylated by SIRT1. Since PPAR*γ* is an important metabolic transcription factor, the activation of PPAR*γ* pathway has a significant protective effect on various acute and chronic injuries of the central nervous system.^[^ ^55^ ^]^ It could be a target for treatment of ischemic cerebrovascular diseases, AD, and PD.^[^ ^56^ ^]^ Therefore, we hypothesized that PPAR*γ* might affect mitochondrial transcription level to promote mitochondrial biogenesis by upregulating the transcription of downstream target gene nuclear respiratory factor 1 (NRF1). To prove this hypothesis, we detected the mRNA expression of NRF1 by qRT‐PCR assay. Surprisingly, the mRNA expression of NRF1 as the downstream gene of PPAR*γ* was upregulated by the pretreatment with CSC NPs as expected, and positively increased with the concentration of CSC NPs (Figure 3g).

Given the crucial role of NRF1 in regulating nuclear genes for mtDNA transcription and replication,^[^ ^57^ ^]^ we further detected the gene level of mitochondrial transcription factor A (TFAM), which encodes a key mitochondrial transcription factor playing significant role in mitochondrial DNA replication.^[^ ^58^ ^]^ Figure S7c of the Supporting Information clearly shows the lever of TFAM in the cells pretreated with 25 µm CSC NPs, which was more than 200 times higher than that of cells treated by 3 mm MPP^+^ for 24 h. These results not only demonstrate the serious damage of mitochondria by MPP^+^ and the protective role of our CSC NPs, but also prove that PPAR*γ* upregulated NRF1 transcription to mediate TFAM transcription.

As mentioned previously, TFAM affects the replication of mtDNA, mitochondrial DNA copy number (mtDNA CN) as an important indicator of mtDNA replication and the number of mitochondria, was examined via qRT‐PCR assay. The level of mtDNA CN in the cells pretreated with 25 µm CSC NPs was 2.6 times higher than that of cells treated with MPP^+^ alone (Figure 3h), which indicates that the decrease of mtDNA CN induced by MPP^+^ was effectively alleviated by CSC NPs. These results collectively prove that the NOX‐like activity of CSC NPs played a vital role in protecting mitochondria through modulating the SIRT1/PGC‐1*α* signaling pathway to mediate transcription of mitochondrial biogenesis (Figure 3i).

### 2.4 Mitochondria‐Targeted CSCCT NPs Improve Neuronal Mitochondrial Biogenesis by an SIRT1‐Dependent Manner

All the previous results demonstrate that CSC NPs can effectively protect mitochondrial function after being phagocytosed by SH‐SY5Y cells in vitro. However, CSC NPs faced with three challenges in targeted delivery into mitochondria, i.e., crossing the blood‐brain barrier (BBB), recognizing neurons and reaching their mitochondrial inner membrane. Most nanoparticles were endocytosed and degraded in lysosomes,^[^ ^59^ ^]^ and did not maximally play the role of protecting mitochondria from damage. To address these issues, we jointly used several strategies including encapsulation of CSC NPs with the membrane of macrophages, modification of membrane with DSPE‐PEG_2000_‐TPP, and transient opening of BBB with noninvasive focused ultrasound (US). It is known that macrophages exhibit the peculiarity of inflammation tropism,^[^ ^60^ ^]^ easy penetration of BBB via interaction between intercellular cell adhesion molecule‐1 (ICAM‐1), and neuronal targeted capability through the interaction between integrin and vascular cell adhesion molecule‐1 (VCAM‐1).^[^ ^61^ ^]^ The noninvasive US can temporarily and reversibly open the BBB to assist the delivery of nanoparticles to the lesion site, which has been successfully used for therapy of various brain diseases.^[^ ^38^, ^39^, ^62^, ^63^ ^]^ The DSPE‐PEG_2000_‐TPP modification can endow the nanoparticles with mitochondria‐targeted capability.

**Figure 4:**
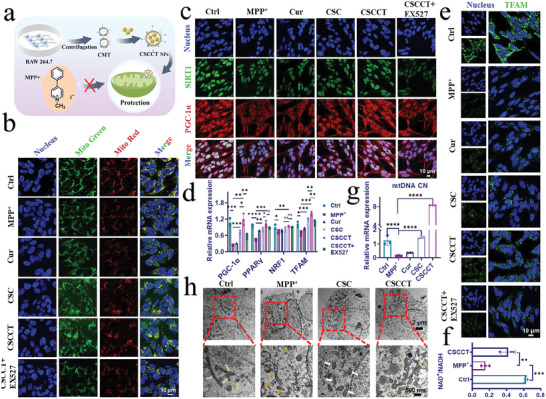
Mitochondria‐targeted CSCCT NPs effectively improved mitochondrial function and biogenesis by activating the pathway of SIRT1/PGC‐1*α* depending on SIRT1. a) Schematic illustration of the preparation of CSCCT NPs and their protection on mitochondria from damage caused by MPP^+^. b) CLSM images indicated total and active mitochondria. Blue: cell nuclei; green: total mitochondria; red: active mitochondria. c) CLSM images of the expressions of SIRT1 and PGC‐1*α* proteins in SH‐SY5Y cells pretreated with Cur, CSC NPs, CSCCT NPs and EX527 (SIRT1 inhibitor), and then incubated with MPP^+^ Green: SIRT1; red: PGC‐1*α*. d) Gene expressions of PGC‐1*α*, PPAR*γ*, NRF1, TFAM in SH‐SY5Y cells analyzed by qRT‐PCR (*n* = 3). e) CLSM images indicated the expression of TFAM in SH‐SY5Y cells received different treatments, blue: cell nuclei; green: TFAM. f) The ratio of NAD^+^ to NADH in SH‐SY5Y cells pretreated with CSCCT NPs (*n* = 3). g) Gene expressions of mitochondrial copy number (mtDNA CN) in SH‐SY5Y cells analyzed by qRT‐PCR (*n* = 3). h) Representative TEM images of mitochondria in SH‐SY5Y cells incubated with MPP^+^ after incubation with CSC and CSCCT NPs. Green arrows indicated intact structure of mitochondria, brown arrows indicated the broken and disappearance of mitochondrial cristae. The two‐sided one‐way ANOVA with a Tukey post hoc analysis was used for comparison among multiple groups. *P < 0.05, **P < 0.01, ***P < 0.001, ****P < 0.0001, ns, not significant.

The membrane of RAW 264.7 cells was isolated and modified with DSPE‐PEG_2000_‐TPP (abbreviated as CMT) for wrapping CSC NPs, and the resulting biomimetic nanoparticles were defined as CSCCT NPs (**Figure** **4**a). The CSC NPs wrapped with macrophage membrane alone denoted as CSCC NPs. The CSCCT NPs had a size of (47.6 ± 7.5) nm determined by TEM, and consisted of tens of individual CSC NPs wrapped in macrophage membrane (Figure S8a,b, Supporting Information). Compared with CSP NPs and CSC NPs, both CSCC and CSCCT NPs had a larger hydrated particle size and a lower zeta potential (Figure S8c,d, Supporting Information), which proves the successful coating of CSC NPs with RAW 264.7 cells membrane. The cytotoxicity of CSCCT NPs toward healthy SH‐SY5Y cells was evaluated by CCK‐8. The result indicates that when the concentration of CSCCT NPs was lower than 50 µm, the cell survival rate was higher than 90% (Figure S9a, Supporting Information). Figure S9b of the Supporting Information shows that with the increase of pretreatment concentration of CSCCT NPs, the viability of SH‐SY5Y cells after treatment with MPP^+^ was gradually increased, higher than that of cells treated with MPP^+^ alone. This result suggests that CSCCT NPs can also protect SH‐SY5Y cells from damage caused by neurotoxins MPP^+^.

Subsequent studies were conducted to investigate the targeting capability of CSCCT NPs to the impaired neuronal cells and their mitochondria. We adopted CLSM to observe the colocalization of mitochondria and biomimetic nanoparticles with different modifications in SH‐SY5Y cells and BV2 cells during different culture time. The CSCC NPs and CSCCT NPs were labeled with FITC, respectively (abbreviated as CSCC‐FITC NPs and CSCCT‐FITC NPs). As displayed in Figures S10 and S11 of the Supporting Information, the uptake of both CSCC‐FITC NPs and CSCCT‐FITC NPs by two kinds of cells reached the maximum after being incubated for 2 h. In addition, CSCCT‐FITC NPs exhibited a better mitochondrial targeting capability to SH‐SY5Y cells than CSCC‐FITC NPs due to the presence of DSPE‐PEG_2000_‐TPP. The overlapping indexes of SH‐SY5Y cells treated with CSCC‐FITC NPs and CSCCT‐FITC NPs for 2 h were 0.7 and 0.9, respectively. However, the overlapping indexes of BV2 cells treated with the same condition were both 0.2. For BV2 cells, neither CSCC‐FITC NPs nor CSCCT‐FITC NPs can target their mitochondria, evidenced by the separated red and green fluorescence. These results suggest that CSCCT NPs can specifically target the mitochondria of neuronal cells. Since most damaged neurons are in an inflammatory environment, we used 0.25 mm MPP^+^ to incubate with SH‐SY5Y cells for 24 h to create and mimic an inflammatory environment, then added CSCC‐FITC NPs and incubated with cells. Figure S12 of the Supporting Information shows that the green fluorescence intensity of cells treated with MPP^+^ was 2.4 times higher than that of without MPP^+^ treatment, which suggests that CSCC‐FITC NPs showed better tropism to the inflammatory neurons. These results further indicate that CSCCT NPs can target the mitochondria of damaged neurons for ameliorating their dysfunction.

Based on the above results, we further explored the targeting mechanism of CSCCT NPs toward neurons. It is well known that the integrin is highly expressed on the surface of macrophage membrane and shows strong affinity to endothelial adhesion molecules such as ICAM‐1 and VCAM‐1.^[^ ^64^ ^]^ Therefore, we used Western blot to explore the surface integrin of cell membrane wrapped on CSC NPs and the expression of VCAM‐1 on SH‐SY5Y cells incubated with MPP^+^ or different concentrations of nanoparticles. Figure S13a of the Supporting Information clearly shows that extraction and extrusion process did not affect the high expression of integrin *α*4 and *β*1 on the macrophage membrane. Different treatments did not influence the expression of VCAM‐1 on the membrane of SH‐SY5Y cells (Figure S13b,c, Supporting Information), demonstrating that CSCCT NPs targeted neurons through the strong interaction between integrin and VCAM‐1.

The above results illustrate the excellent mitochondria‐targeted capability of CSCCT NPs and suggest their better performance in amelioration of mitochondrial dysfunction. To demonstrate the effect of CSCCT NPs on SIRT1, we used EX527 as an inhibitor to suppress the expression of SIRT1. SH‐SY5Y cells were pretreated with Cur (25 µm), CSC NPs (25 µm), CSCCT NPs (25 µm), CSCCT NPs (25 µm) after incubation with EX527 (10 µm) for 12 h, respectively, and then incubated with 3 mm MPP^+^ for 24 h. The total mitochondria (green fluorescence) and active mitochondria (red fluorescence) were observed after being stained with mitochondrial trackers (Figure 4b). The results clearly show that CSCCT NPs had a better mitochondrial protective effect than CSC NPs and Cur after quantitative analysis (Figure S14, Supporting Information). However, this protective effect was attenuated by SIRT1 inhibitor EX527. The immunofluorescence assay was also used to detect the expression of SIRT1 and PGC‐1*α* in the SH‐SY5Y cells (Figure 4c). Similar to the previous results, compared with the cells pretreated with CSC NPs, the SH‐SY5Y cells treated with CSCCT NPs showed excellent colocalization of PGC‐1*α* and nucleus (Figure S15a, Supporting Information). The expression of SIRT1 and PGC‐1*α* was significantly decreased after the cells were treated with MPP^+^ alone. Treatment of cells with Cur alone slightly influenced the expression of SIRT1 and PGC‐1*α*. By contrast, both CSC NPs and CSCCT NPs showed super protective effect on SIRT1 and PGC‐1*α*, both which were notably increased in the treated cells in comparison with the cells treated with MPP^+^ alone (Figure S15b, Supporting Information). Moreover, CSCCT NPs exhibited better protection on cells than CSC NPs due to their targeting capability. However, their protective effect was attenuated by EX527, evidenced by the drastic decrease in SIRT1 and PGC‐1*α*. These results indicate that the protective role of CSCCT NPs on neuronal mitochondria is dependent on SIRT1, and PGC‐1*α* is a downstream protein of SIRT1.

Based on the above results, we applied qRT‐PCR for measuring the expression of PGC‐1*α* and its downstream related gene to verify the mitochondrial biogenesis and its related SIRT1/PGC‐1*α* pathway. When the cells were pretreated with CSCCT NPs, the expression of PGC‐1*α* was increased, leading to an increase in the mRNA expressions of PPAR*γ*, NRF1, and TFAM, which were inhibited by the addition of inhibitor EX527 (Figure 4d). These results suggest the upregulation of downstream genes of PGC‐1*α* by our nanoparticles.

To accurately demonstrate the protective effect of nanoparticles on mitochondrial respiratory chain, we carried out Western blot analysis of Ndufs1. Figure S16 of the Supporting Information shows that CSCCT NPs exhibited the best protective effect in comparison with Cur and CSC NPs. Moreover, we performed immunofluorescence staining of TFAM to determine mitochondrial biogenesis and transcription. After incubation of SH‐SY5Y cells with MPP^+^ for 24 h, the fluorescence intensity of TFAM was largely weakened (Figure 4e; Figure S17, Supporting Information). However, if SH‐SY5Y cells were pretreated with Cur, CSC NPs, and CSCCT NPs for 2 h before treatment with MPP^+^, the fluorescence intensity of TFAM was partially maintained, in which CSCCT NPs showed the best protective effect that could be inhibited by EX527.

These above results prove that the recovery of mitochondrial respiratory chain and transcription was mediated and regulated by SIRT1/PGC‐1*α* and the downstream factors. The activities and expression of SIRT1 are obligatorily dependent on cellular mitochondrial NAD^+^, which is a vital cofactor regulating metabolic homeostasis, and is the rate‐limiting substrate of sirtuin deacetylase.^[^ ^54^ ^]^ It is crucial to further investigate the changes of intracellular NAD^+^ via the NAD^+^/NADH assay kit. Figure 4f shows that mitochondria‐targeted CSCCT NPs improved the ratio of NAD^+^ to NADH to activate the expression of SIRT1 and enhance the deacetylation of PGC‐1*α*, which triggered a positive cascade of mitochondrial biogenesis.

In the next, several methods were used to detect the indicators of mitochondria biogenesis and characterize the subsequent beneficial effects from improved mitochondria biogenesis. We first measured the mRNA expressions of endogenous antioxidative enzymes, including Gpx4, catalase (CAT), and SOD2, to evaluate the changes of cellular antioxidative capability after improvement of mitochondrial biogenesis. The results clearly demonstrate that the genes of Gpx4, CAT, and SOD2 in the SH‐SY5Y cells pretreated with CSCCT NPs (25 µm for 2 h) maximally restored the cellular antioxidative capability compared with the cells pretreated with Cur (25 µm for 2 h) and CSC NPs (25 µm for 2 h) (Figure S18a–c, Supporting Information). When the inhibitor EX527 was added with CSCCT NPs to SH‐SY5Y cells, the mRNA expressions of those antioxidative enzymes were notably decreased, suggesting that the function of these antioxidative enzymes was largely dependent on mitochondrial biogenesis.

We further used the CLSM to characterize the generation of ROS in SH‐SY5Y cells, which were preincubated with Cur, CSC NPs, or CSCCT NPs for 2 h, and then incubated with MPP^+^ for 24 h. The CLSM images and quantitative analysis show that CSCCT NPs greatly prevented ROS production in mitochondria caused by MPP^+^ damage (Figure S19, Supporting Information). In addition, MPP^+^ reduced the MMP with the attenuation of red fluorescence from JC‐1 aggregates and the enhancement of green fluorescence from JC‐1 monomers, which was also prevented by CSCCT NPs (Figure S20, Supporting Information). The release of large amounts of Cyt c after mitochondrial damage can initiate the apoptosis of cells, and immunofluorescence staining was used to detect the release of Cyt c. The least release of Cyt c in the cells pretreated with CSCCT NPs demonstrates their best protective effect, indicating the less cleavage of caspase 3 and less apoptosis of cells (Figure S21, Supporting Information). The above results prove that the mitochondrial damage caused by MPP^+^ can be effectively prevented via CSCCT NPs. The efficacy of CSCCT NPs was further demonstrated by their effects on neuronal mitochondrial biogenesis and function. qRT‐PCR was carried out to determine the mRNA expression of mtDNA CN in the SH‐SY5Y cells received different treatments, because mtDNA CN indicates the health of mitochondria. Surprisingly, the expression level of mtDNA CN in the cells pretreated with CSCCT NPs was more than 50 times higher than that in the cells treated with MPP^+^ alone, and eight times more than that of normal cells without any treatment (Figure 4g), which indicates that the mitochondria in SH‐SY5Y cells became more active and healthier with the protection of CSCCT NPs even in the presence of MPP^+^.

To further demonstrate the amelioration of mitochondria, the mitochondrial structure of untreated SH‐SY5Y cells and the cells pretreated with CSC NPs and CSCCT NPs, and then incubated with MPP^+^ was observed via biological TEM. In the healthy cells, their mitochondrial structure was intact and rod‐shaped. However, the mitochondria of MPP^+^‐treated cells were seriously damaged, and they were significantly shorter, rounder, even expanded and cleaved. After pretreated with CSC NPs and CSCCT NPs, the morphology of mitochondria tended to be normal. The mitochondrial cristae of cells pretreated with CSCCT NPs were more clear and complete, which were closer to that of healthy mitochondria (Figure 4h). The results suggest that mitochondria‐targeted CSCCT NPs can more effectively prevent mitochondrial damage and improve mitochondrial function.

After assessing the improvement of mitochondrial structure and function, we finally assessed the redox balance in the whole cellular environment. We used the fluorescence probe DCFH‐DA to measure the total ROS generated in SH‐SY5Y cells received different treatments. Nonfluorescent DCFH‐DA can be oxidized into DFH to emit strong green fluorescence under excitation. Since MPP^+^ destroyed the mitochondrial respiratory chain and led to the leakage of free electrons, the amount of intracellular ROS was increased. Figure S22 of the Supporting Information shows the strong green fluorescence of probe in the cells treated with MPP^+^ due to the destroy of mitochondrial respiratory chain, which was drastically attenuated by pretreating cells with nanoparticles, particularly with CSCCT NPs.

In addition to antioxidative enzymes, intracellular small molecules also play vital role in maintaining the redox balance in cells. One typical small molecule is the reduced glutathione (GSH), which is a key antioxidant accounting for 90–95% of total glutathione, and serves as the main source of sulfhydryl groups in proteins. GSH can be oxidized into glutathione disulfide (GSSG) by ROS. We measured the ratio of GSH to GSSG using an assay kit, and found that the pretreatment of SH‐SY5Y cells with CSC NPs and CSCCT NPs restored the GSH/GSSG ratio to the normal level (Figure S23, Supporting Information). All these results collectively suggest that CSCCT NPs can prevent mitochondria from damage to maintain the redox balance in the cells by promoting SIRT1‐dependent mitochondrial biogenesis.

### 2.5 CSCCT NPs Are Efficiently Delivered into the Neurons of MPTP‐Induced PD Mice

In vitro experiments comprehensively demonstrate that CSCCT NPs can target mitochondria of neurons in an inflammatory environment to improve their mitochondrial biogenesis. To efficiently deliver these nanoparticles to the lesion area in the brain, we jointly used the focused US to overcome the BBB issue in vivo. We previously reported that focused US can temporarily and reversibly open the BBB to assist the delivery of nanoparticles into the brain parenchyma^[^ ^65^, ^66^ ^]^ and proved that Cu_2‐_ *_x_* Se nanoparticles exhibited super performance for photoacoustic (PA) imaging due to their strong local surface plasmon resonance.^[^ ^67^, ^68^ ^]^ Similarly, we performed PA imaging to determine the accumulation of CSCCT NPs in the brain. First, we injected Evans blue (EB) through the tail vein of mice after sonicating the corpus striatum of right brain, and then resected the whole brain of mice to confirm that focused US can open the BBB safely to allow EB reaching the deep tissue (Figure S24, Supporting Information). We intravenously injected CSCCT NPs solution into mice through the tail vein and collected the PA signals at different time points of post‐injection. Without the application of focused US, the intensity of PA signal in the brain parenchyma was slightly increased and reached the maximum at 2 h after injection (Figure S25a, Supporting Information). By contrast, application of sonication after injection of microbubble (MB) and CSCCT NPs resulted in significant enhancement of PA signal in the right brain, which reached the maximum at 2 h after injection, followed by gradual decrease (Figure S25b,c, Supporting Information). The strong PA signal was due to the synergistic contribution of focused US and the strong affinity between the macrophage membrane on the surface of CSCCT NPs and vascular endothelial cells. These results suggest that CSCCT NPs were efficiently delivered to the striatum and substantia nigra with assistance of focused US, which laid the foundation for subsequent therapy for PD in vivo.

To investigate the capability of CSCCT NPs targeting neurons at the inflammatory site, the right substantia nigra of brain was sliced for observation after the three groups of mice were administrated with FITC‐labeled nanoparticles, respectively, i.e., I) healthy mice injected with CSCCT‐FITC NPs through their tail veins after US sonication (Ctrl + US + CSCCT‐FITC group); II) MPTP‐induced PD mice injected with CSCC‐FITC NPs through their tail veins after US sonication (PD + US + CSCC‐FITC group); and III) MPTP‐induced PD mice injected with CSCCT‐FITC NPs through their tail veins after US sonication (PD + US + CSCCT‐FITC group). The tyrosine hydroxylase (TH) in the brain tissue slices were stained by immunofluorescence assay to evaluate the mitochondrial targeting in neuronal cells. Figure S26 of the Supporting Information shows that compared with PD + US + CSCCT‐FITC group of mice, the mean fluorescence intensity of green fluorescence in the Ctrl + US + CSCCT‐FITC group of mice was much lower, which is due to the lack of inflammatory environment in the brain of healthy mice. Meanwhile, stronger green fluorescence in PD + US + CSCC‐FITC group of mice was distributed in the periphery of dopaminergic neurons in PD mice. These results demonstrate that CSCCT NPs can target neurons in MPTP‐induced PD mice.

### 2.6 Therapeutic Efficacy of CSCCT NPs on MPTP‐Induced PD Mice

**Figure 5:**
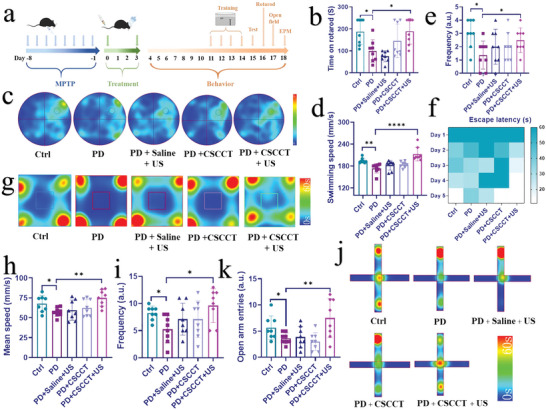
Therapy of MPTP‐induced PD mice with biomimetic CSCCT NPs. a) Schematic illustration of MPTP‐induced PD mice, their therapy with CSCCT NPs and the course of behavior test. b) Rotatory‐rod test of mice from the Ctrl group, PD group, PD + Saline + US group, PD + CSCCT group, and PD + CSCCT + US group (*n* = 8). c) Morris water maze test from five groups of mice. Heat map of tracking path and parameter analyses showed changes in d) the swimming speed, e) the frequency through the target quadrant (*n* = 8), f) heat map of escape latency in successive five days. g) Open field test. Heat map of tracking path and parameter analyses showed changes in h) the mean speed, i) central zone transition number from five groups of mice (*n* = 8). j) Elevated plus‐maze (EPM) test. Heat map of tracking path and parameter analyses showed changes in k) the frequency of open arm entries from five groups of mice (*n* = 8). Data represented the mean ± SD (*n* = 8). The two‐sided one‐way ANOVA with a Tukey post hoc analysis was used for comparison among multiple groups. *P < 0.05, **P < 0.01, ***P < 0.001, ****P < 0.0001, ns, not significant.

The previous in vitro results indicate that CSCCT NPs can effectively improve mitochondrial dysfunction caused by MPP^+^, and they can be effectively delivered into brain with assistance of focused US. To investigate the protective and therapeutic effects of CSCCT NPs in vivo, we established a mouse model of PD induced by MPTP for therapy and subsequent behavioral tests (**Figure** **5**a). The mice were randomly divided into five groups (*n* = 8), i.e., 1) normal wild type C57BL/6J mice (Ctrl group), 2) PD mice without any intervention (PD group), 3) PD mice injected with saline through their tail veins after US sonication (PD + Saline + US group), 4) PD mice only intravenously injected with CSCCT NPs (2 mg kg^−1^) (PD + CSCCT group), and 5) PD mice intravenously injected with CSCCT NPs (2 mg kg^−1^) after US sonication (PD + CSCCT + US group).

It is well known that Parkinson's symptoms include motor symptoms (e.g., quiescent tremors and postural balance disorders) and non‐motor symptoms (e.g., anxiety and cognition impairment).^[^ ^69^ ^]^ In the aspect of motor function, rotatory‐rod test was first carried out to test the mouse capability of keeping balance. The time of mice staying on the rod in the PD group (99.125 s) was significantly shorter than that of mice from the PD + CSCCT + US group (189.125 s), which was similar to that of normal group (187.625 s) (Figure 5b). This result indicates that the PD mice treated with CSCCT NPs and US regained the best athletic ability. Afterwards, we used a water maze to test the ability of five groups of mice to learn and remember spatial location and orientation. The heat map and actual trajectory of their representative swimming paths on day 4 post‐training (Figure 5c; Figure S27a, Supporting Information) show that the mice from the Ctrl group and PD + CSCCT + US group found the escape platform more easily and accurately than the mice from the PD group, which preferred to stay in the corners of the four quadrants. The mice in the PD + CSCCT + US group swam faster (212.85 mm s^−1^) than the mice in the PD group (177.17 mm s^−1^) (Figure 5d). They searched for the escape platform more frequently than other groups of mice (Figure 5e), indicating that the PD mice treated with PD + CSCCT + US had better learning and memory ability. Besides, the escape latency collected in five consecutive days was analyzed into heat map and histogram (Figure 5f; Figure S28, Supporting Information), which clearly show that the escape latency of mice in the PD + CSCCT + US group became shorter and shorter (from 60 s to 11.03 s) with the increase of forced swimming time. However, there was no significant change in the escape latency for the mice from the PD group. These results prove that the mice in the PD + CSCCT + US group showed excellent learning and exploration ability after training.

The above results indicate that CSCCT NPs had a great therapeutic effect on the motor and cognition ability of PD mice. In terms of nonmotor symptoms, the open field was initially used to observe voluntary behavior, exploratory behavior and anxiety in PD mice after different treatments. Specifically, the heat map and path diagram of their representative paths indicate that the mice from the PD + CSCCT + US group spent less time in the corner and more time moving between the central and peripheral areas (Figure 5g; Figure S27b, Supporting Information), suggesting that they had a greater willingness of independent exploration. The augment of mean speed and center entrance numbers demonstrate that the nerves and independent exploration dysfunction caused by MPTP were significantly ameliorated in the mice from the PD + CSCCT + US group (Figure 5h,i). The elevated plus‐maze was further applied for detecting the anxiety of mice from five groups after treatments. The principle is that the mice usually have the curiosity of exploration for the new things (open arm), while they have the nature of staying in darkness (close arm), and thus exhibit the conflict behavior of exploration and avoidance. The heat map and path of their representative paths indicates that PD mice induced by MPTP obviously exhibited anxious behavior with long stagnation in the corner of the close arm, but the mice in the PD + CSCCT + US group explored the open arm area as frequently as the mice in the Ctrl group (Figure 5j; Figure S27c, Supporting Information). The open arm entries and the proportion of the open arm residence time in the total time for the mice from the PD + CSCCT + US group (7.5, 23.47 s) were increased significantly compared with that of the PD mice (3.6, 11.22 s) (Figure 5k; Figure S29, Supporting Information). The results of EMP reveal that the mice from the PD + CSCCT + US group had less anxiety‐like behavior. These behavioral tests and results collectively indicate the excellent therapeutic efficacy of our CSCCT NPs in restoring the motor capability and memory function, and overcoming anxiety of MPTP‐induced PD mice.

**Figure 6:**
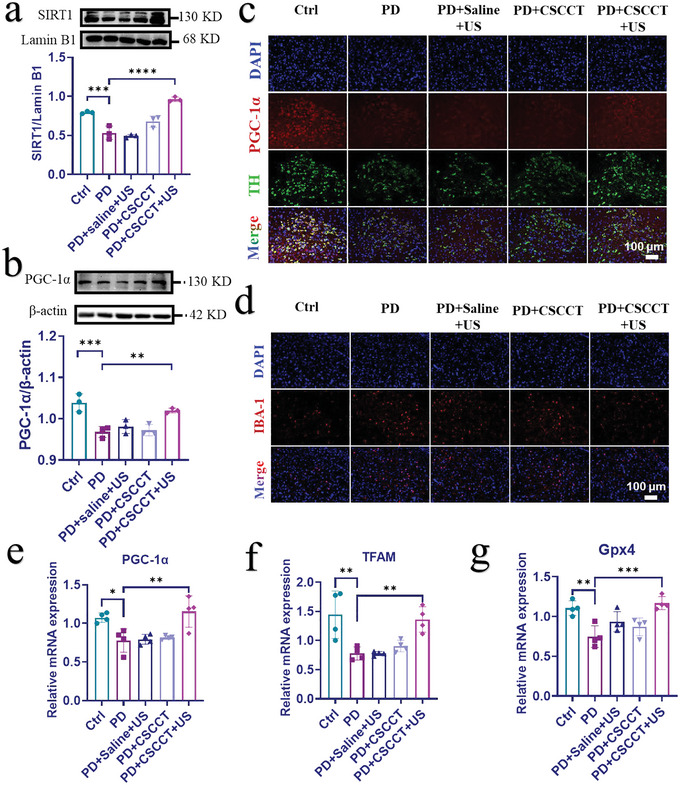
Therapeutic efficacy of CSCCT NPs on MPTP‐induced PD mice. a,b) Western blot and quantitative analyses of SIRT1 and PGC‐1*α* proteins from five groups of mice: 1) Ctrl group, 2) PD group, 3) PD + Saline + US group, 4) PD + CSCCT group, and 5) PD + CSCCT + US group (*n* = 3). c,d) Immunofluorescence staining of, PGC‐1*α*, TH, and IBA‐1 from the right substantia nigra of mice from the above five groups. e–g) qRT‐PCR assay of the relative gene expressions of PGC‐1*α*, TFAM, and Gpx4 in the right substantia nigra of mice from the above five groups (*n* = 4). The two‐sided one‐way ANOVA with a Tukey post hoc analysis was used for comparison among multiple groups. *P < 0.05, **P < 0.01, ***P < 0.001, ****P < 0.0001, ns, not significant.

The therapeutic effect of our CSCCT NPs on PD mice was further investigated at protein levels. Compared with the untreated PD mice, the PGC‐1*α* and SIRT1 proteins in the right substantia nigra from the mice in the PD + CSCCT + US group were clearly increased and close to that of normal mice, which indicate the recovery of neuronal mitochondrial biogenesis in the substantia nigra (**Figure** **6**a,b). The expression of Ndufs1 also suggests that the mitochondrial respiratory chain in the mice from PD + US + CSCCT group worked well, which was similar to that of mice from the Ctrl group (Figure S30, Supporting Information). In addition, immunofluorescence staining was used to monitor the changes of PGC‐1*α*, TH, and ionized calcium‐binding adaptor protein 1 (IBA‐1). TH is a rate‐limiting factor in the biosynthesis of catecholamines such as dopamine, epinephrine, and norepinephrine.^[^ ^70^ ^]^ IBA‐1 is a marker of microglial activation, which can reflect the response of mice to the stress and injury in the central nervous system.^[^ ^71^ ^]^ As shown in Figure 6c and Figure S31 (Supporting Information), compared with the healthy mice in the Ctrl group, both the intensity of PGC‐1*α* and TH in the PD mice were significantly decreased. By contrast, both them were recovered and well overlapped in the mice from the PD + CSCCT + US group. Different from the mice in the PD group, the fluorescence intensity of IBA‐1 in the mice from the PD + CSCCT + US group was reduced to the normal level of healthy mice (Figure 6d; Figure S31, Supporting Information), suggesting that increased dopaminergic neurons and decreased activation of microglia.

At the genetic level, PGC‐1*α*, TFAM, and Gpx4 in the right substantia nigra of five groups of mice were finally measured by qRT‐PCR. Figure 6e–g clearly shows that PGC‐1*α*, TFAM, and Gpx4 in the PD mice treated with CSCCT NPs and US were the most abundant among the three treatment groups of mice, which proves that mitochondrial biogenesis was ameliorated by CSCCT NPs and the antioxidant enzyme Gpx4 in mitochondria was restored to the normal level. These results indicate that our CSCCT NPs have broad applications in the neuroprotection and treatment for PD.

The toxicity of CSCCT NPs in vivo was assessed by hematoxylin–eosin (H&E) staining of major organs (i.e., heart, liver, spleen, lung, and kidney) of mice after various treatments. Figure S32 of the Supporting Information shows that CSCCT NPs did not cause damage or side effects on the major organs of mice, which indicate CSCCT NPs have good in vivo biocompatibility.

## 3 Conclusion

In summary, ultrasmall Cu_2‐_ *_x_* Se‐based mitochondria‐targeted biomimetic nanoparticles (CSCCT NPs) can be efficiently accumulated in the neuronal mitochondria to promote the expression of SIRT1, which deacetylates PGC‐1*α* to modulate neuronal mitochondrial biogenesis via upregulating the level of NAD^+^ in mitochondria. CSCCT NPs can target the inflammatory neurons and their mitochondria to regulate the pathway of NAD^+^/SIRT1/PGC‐1*α*/PPAR*γ*/NRF1/TFAM for improving mitochondrial biogenesis. With the assistance of focused ultrasound, CSCCT NPs can be more efficiently delivered to the brain to alleviate neuronal mitochondrial dysfunction caused by MPTP, and significantly improve the motor ability and emotional behavior of PD mice. This work demonstrates the great potential of targeting neuronal mitochondrial biogenesis to improve mitochondrial dysfunction, which opens a new window for the treatment of Parkinson's and other mitochondrial diseases.

## 4 Experimental Section

### Materials

CuCl_2_·2H_2_O (>99%), NaBH_4_ (>99%), selenium powder (−100 mesh, ≥99.5%), polyvinylpyrrolidone (PVP, >99%), and MPP^+^ iodide were purchased from Sigma‐Aldrich. Curcumin was purchased from Sinopharmatic Chemical Reagent Suzhou Co., Ltd. EX527, SIRT1 inhibitor, was purchased from Selleck Co., Ltd. MPTP hydrochloride was purchased from Meilun Biotechnology Co., Ltd. N_2_ was purchased from Wu Jiang Guo Rong Co., Ltd. Milli‐Q water (18 MΩ cm) was used in the experiments. All chemicals and reagents were used without any further purification.

### Characterization

The EDS, TEM, and HRTEM images were captured using the high‐resolution field emission transmission electron microscope Talos F200s G2 operating at an acceleration voltage of 200 kV. DLS and *ζ* potential measurements were conducted at room temperature on a Malvern Zetasizer Nano ZS90 equipped with a solid‐state He‐Ne laser (*λ* = 633 nm). The crystal structure of CSP NPs was characterized with a Shimadzu XRD‐6000 X‐ray diffractometer equipped with Cu K*α*1 radiation (*λ* = 0.15 406 nm). The UV–vis–NIR spectra were collected on a PerkinElmer Lambda 750 UV–vis–NIR spectrophotometer. FTIR was tested by the Thermo Scientific NICOLET iS 50 conventional Fourier infrared spectrometer. The TGA of Cur, CSP, and CSC NPs was characterized by the Thermo STA 499 F3 jupiter. The copper ions were determined by the inductively coupled plasma mass spectrometry (ICP‐MS, Thermo, ELEMENT 2).

### Synthesis of Ultrasmall Cu_2‐_ *_x_* Se‐PVP Nanoparticles (Abbreviated as CSP NPs)

CSP NPs were prepared by one‐pot approach as previously reported.^[^ ^40^ ^]^ 39.48 mg selenium powder and 56.75 mg NaBH_4_ were added into 50 mL Milli‐Q H_2_O, which was bubbled with nitrogen to remove oxygen under magnetic stirring. The selenium powder and NaBH_4_ were gradually dissolved and reduced under nitrogen protection. Then 85 mg CuCl_2_·2H_2_O and 200 mg PVP (*M*_w_ = 8000) were dissolved in another 25 mL Milli‐Q H_2_O under magnetic stirring and nitrogen protection at room temperature until the solids were completely dissolved. The half of the selenium precursor solution (V = 25 mL) was mixed with copper precursor solution under nitrogen atmosphere. The mixture solution turned into a brownish black solution immediately, which was stirred for 2 h at room temperature under nitrogen protection. The final solution was centrifuged for 10 min at a speed of 10 000 rpm, the supernatant was collected and stored at 4 °C for use.

### Synthesis of Cu_2‐_ *_x_* Se‐PVP‐Curcumin Nanoparticles (Abbreviated as CSC NPs)

The stored CSP NPs was ultrafiltrated for ten times (10 min for each time) at 4500 rpm by using a 30 KDa ultrafiltration tube to remove excessive PVP. 1 mL resultant CSP NPs solution (4 mg mL^−1^) was diluted with 4 mL dimethyl sulfoxide (DMSO) and 3 mL H_2_O (V:V = 1:1). 12 mg curcumin (Cur) powder was first dissolved in 400 µL DMSO and then dropwised into the CSP NPs solution to result in a pale brown mixture. After vigorous stirring for 4 h at room temperature, the solution was transferred into a universal dialysis tube (cut‐off molecule weight = 8000–14 000 Da) and dialyzed with H_2_O for 24 h. The dialysis water was renewed every 2 h. The whole solution was centrifuged at 7000 rpm for 10 min, and the supernatant was centrifuged for 30 min at 3500 rpm with 30 KDa ultrafiltration tube, and the concentrated CSC NPs solution was stored at 4 °C for use.

### Modification of Cell Membrane with DSPE‐PEG_2000_‐TPP (Abbreviated as CMT)

The cell membrane was extracted according to a previously reported method.^[^ ^44^ ^]^ RAW 264.7 cells were washed with PBS three times and harvested by centifuging at 1000 rpm for 5 min, then these cells were resuspended in a cold Tris buffer (pH = 7.4) (consist of 1 × ethylenediamine tetra‐acetic acid (EDTA)‐free protease inhibitor, 10 mm MgCl_2_, and 10 mm Tris) for 1 h at 4 °C. The suspension was sonicated for 15 min in an Ultrasonic Cell Disruption System with an ice‐bath, followed by centrifugation at 10 000 × *g* for 10 min at 4 °C (Allegra 64R, Beckman Coulter, Inc.). The obtained supernatants were centrifuged at 100 000 × *g* for 1 h (Optima L‐100XP, Beckman Coulter, Inc.) to obtain the cell membrane (CMs) debris. After the CMs were resuspended in PBS solutoin containing EDTA‐free protease inhibitor, 0.2 mL DSPE‐PEG_2000_‐TPP (25 mg mL^−1^) was added into 4 mL CMs (0.25 mg mL^−1^), followed by sonication for 15 min in an Ultrasonic Cell Disruption System with an ice‐bath. Subsequently, the gradient centrifugation was used to collect the CMT, which was resuspended and stored at −80 °C for use.

### Synthesis of CSCCT NPs

The above modified CMT was mixed with CSC NPs solution and extruded for ten cycles through 400 nm polycarbonate membrane to obtain the CSCCT NPs, which were stored at 4 °C for use.

### Assay of Total Antioxidative capability of CSC NPs

The total antioxidative capability of CSC NPs was measured by using ABTS as a probe (Cat. no. S0121, Beyotime). Briefly, 6.25, 12.5, 25, and 50 µm CSC NPs were added into 96‐well plate (10 µL per well). Then 190 ABTS working solution was added and mixed with CSC NPs solutions, which were incubated at 37 °C for 1 h. The absorbance of mixed solution (A414 nm) was recorded on an UV–vis–NIR spectrophotometer (PerkinElmer EnSpire, Singapore).

### Enzyme‐Like Activity of CSC NPs

The Cur, CSP NPs and CSC NPs of 12.5, 25, and 50 µm were prepared. According to the instructions of SOD enzyme activity detection kit (Cat. no. BC0175, Solarbio) and NOX enzyme activity detection kit (Cat. no. BC0635, Solarbio), respectively, and the absorption of mixture solutions was detected by 96‐well plate and microplate reader.

### Cell Culture

RAW 264.7 cells were cultured in the Dulbecco's modified Eagle Medium (HyClone). SH‐SY5Y cells were cultured in the mixture of Minimum Essential Medium (MEM, Gibco) and F12 with an equal proportion (MEM/F‐12, Gibco). All the media contained 10% fetal bovine serum (Gibco) and 1% antibiotics (penicillin/streptomycin), and all cells were cultured at 37 °C in a 5% CO_2_ atmosphere.

### Assay of Cell Toxicity

1 × 10^4^ SH‐SY5Y cells were seeded into a 96‐well plate and cultured for 24 h, and then incubated with different concentrations of CSC NPs (0, 1.56, 3.125, 6.25, 12.5, 25, 50, 100, 200 µm) for 2 h or different concentrations of MPP^+^ (0.125, 0.25, 0.5, 1, 2, 3 mm) for 24 h. 10 µL of CCK‐8 solution (Enhanced Cell Counting Kit‐8, Cat. no. C0043, Beyontime) was added to each well and incubated for additional 2 h at 37 °C. The absorbance of formazan at 450 nm was measured using a microplate reader (PerkinElmer EnSpire, Singapore). The relative cell viabilities were calculated by the following formula:

Cell viability (%) = [A(sample)–A(blank)]/[A(control)–A(blank)] × 100%, where A(sample) was the mean absorbance of the treated cells, A(control) was the mean absorbance of untreated cells, and A(blank) was the mean absorbance of blank solution. When the protective effects of CSC NPs were evaluated, the SH‐SY5Y cells were pretreated with 6.25, 12, 5, 25, and 50 µm of the CSC NPs for 2 h, followed by culturing with fresh medium containing 3 mm MPP^+^ for another 24 h. The morphology of cells from the Ctrl group, MPP^+^ group and CSC NPs group were observed with an inverted microscope (Olympus, Japan).

### Detection of ATP Content

The relative ATP content was measured using CellTiter‐Lumi Luminescent Cell Viability Assay Kit (Cat. no. C0068M, Beyotime). Briefly, SH‐SY5Y cells were seeded on a black and untransparent 96‐well plate with a density of 8 × 10^3^–1 × 10^4^ cells per well and cultured for 24 h. Then the cells were respectively incubated with different concentrations of CSC NPs (6.25, 12.5, 25 µm) for 2 h, MEM/F‐12 medium containing 3 mm MPP^+^ was added to the cells for another 24 h, 100 µL of Celltiter‐Lumi luminescence assay reagent was added to each well, then oscillated for 2 min at room temperature to promote cell lysis. Finally, the chemiluminescence was detected using a multifunctional microplate reader (PerkinElmer EnSpire, Singapore).

### Determination of Intracellular Mitochondrial Hydroxyl Radical and Total ROS

The generation of hydroxyl radical (·OH) in mitochondria was monitored by Cell Meter Mitochondrial Hydroxyl Radical Detection Kit (MitoROS, Cat. no. 16055, AAT Bioquest). Briefly, SH‐SY5Y cells were seeded in 8‐well confocal dishes with a density of 8 × 10^3^–1 × 10^4^ cells per well and cultured for 24 h. Then the cells were respectively incubated with CSC NPs (6.25, 12.5, and 25 µm), Cur (25 µm), and CSCCT NPs (25 µm) for 2 h. After that, fresh culture medium containing 3 mm MPP^+^ was added to the dishes for another 24 h, washed twice with PBS. For detection the ROS generated in mitochondria, 100 µL MitoROS working solution was added into cells and cultured for 60 min at 37 °C. The cells were then slowly washed three times with culture medium, the fluorescence images were collected with a CLSM (FV1200, Olympus, Japan, excitation: 540 nm, emission: 590 nm, cut off: 570 nm). For the detection of total ROS, 10 µm DCFH‐DA was added into cells and cultured at 37 °C for 30 min. The cells were then slowly washed three times with culture medium, and the nuclei were stained with Hoechst 33342 (5 µg mL^−1^) for 10 min at 37 °C. The fluorescence images were collected with a CLSM (FV1200, Olympus, Japan, excitation: 488 nm, emission: 520 nm).

### Staining of Total and Active Mitochondria

SH‐SY5Y cells were seeded on 8‐well confocal dishes with a density of 8 × 10^3^–1 × 10^4^ cells per well and cultured for 24 h. SH‐SY5Y cells were incubated with 100 µL MitoTracker Red CMXRos working solution (Cat. no. C1049B, Beyotime) for 30 min at 37 °C. After being washed three times with fresh medium, then they were stained with 100 µL MitoTracker Green working solution (Cat. no. C1048, Beyotime) at 37 °C for 30 min. Then, the cells were stained with 10 µg mL^−1^ Hoechst 33342 for 10 min at room temperature, washed three times with PBS, and examined under a CLSM (FV1200, Olympus, Japan, excitation: 549, 488, and 405 nm, emission: 599, 516, and 461 nm, respectively).

### Assay of MMP

The MMP was measured using JC‐1 as a fluorescence probe (Cat. no. C2005, Beyotime). SH‐SY5Y cells (8 × 10^3^–1 × 10^4^ cells per well) were seeded in 8‐well confocal dishes for 24 h. After being incubated with CSC NPs (25 µm), Cur (25 µm), and CSCCT (25 µm) for 2 h, they were further cultured with MEM/F‐12 medium containing 3 mm MPP^+^ for 24 h. The cells were washed twice with PBS, then stained with JC‐1 (5 µg mL^−1^) for 15 min at 37 °C. The fluorescence images were characterized by CLSM (FV1200, Olympus, Japan, excitation: 488 and 559 nm, emission: 529 and 590 nm, respectively).

### Assay of the Ratio of GSH to GSSG

The ratio of reduced GSH to oxidized GSSG was measured by GSH and GSSG Assay Kit (Cat. no. S0053, Beyotime). 1 × 10^4^–2 × 10^4^ SH‐SY5Y cells were seeded in 60 mm dishes and cultured for 24 h. After being incubated with CSC NPs (25 µm), and CSCCT (25 µm) for 2 h, they were further cultured with MEM/F‐12 medium containing 3 mm MPP^+^ for 24 h. Then the cells were collected and washed with PBS, the protein removal reagent was added to the cells and thoroughly mixed, which were frozen and thawed twice in liquid nitrogen and 37 °C water bath. Then the solution was centrifuged at 5600 rpm at 4 °C for 10 min. Part of the supernatant was added to the total GSH detection regent for the determination of total GSH. GSH scavenging reagent was added into another part of the supernatant and mixed for 1 h at 25 °C for GSSG determination. The content of GSH was obtained by subtracting GSSG from total glutathione content.

### Assay of the Ratio of NAD^+^ to NADH

The ratio between oxidized coenzyme I (NAD^+^) and reduced coenzyme I (NADH) was measured by NAD^+^/NADH Assay Kit with WST‐8 (Cat. no. S0175, Beyotime). 1 × 10^4^–2 × 10^4^ SH‐SY5Y cells were seeded in 60 mm dishes and cultured for 24 h. After being incubated with CSC NPs (25 µm), and CSCCT (25 µm) for 2 h, they were further cultured with MEM/F‐12 medium containing 3 mm MPP^+^ for 24 h. The cells was collected and washed with PBS, the extracted solution of NAD^+^/NADH was added, mixed, and lysed at 4 °C for 30 min, then centrifuged at 6720 rpm for 30 min at 4 °C, the supernatant was collected for later use. The total amount of NAD^+^ and NADH was determined by adding alcohol dehydrogenase working solution and chromogenic solution into the previous supernatant, incubated at 37 °C for 30 min in the darkness, and finally measured the absorbance at 450 nm. For the determination of the amount of NADH, the supernatant was incubated in 60 °C water bath for 1 h to decompose NAD^+^. Then, the above procedure was used to measure the total amount of NAD^+^ and NADH.

### Assay of Neuronal Mitochondrial Targeting Capability In Vitro

SH‐SY5Y cells or BV2 cells (8 × 10^3^–1 × 10^4^ cells per well) were seeded in 8‐well confocal dishes and cultured for 24 h, respectively. After being incubated with CSCC‐FITC NPs or CSCCT‐FITC NPs for 1, 2, 4 h, cells were incubated with 100 µL MitoTracker Red CMXRos working solution (Cat. no. C1049B, Beyotime) for 30 min at 37 °C. After washing three times with fresh medium, the fluorescence of cells were observed with a CLSM (FV1200, Olympus, Japan, excitation: 549 nm, 488 nm, emission: 599 nm and 516 nm, respectively).

### Assay of Inflammatory Tropism In Vitro

SH‐SY5Y cells (8 × 10^3^–1 × 10^4^ cells per well) were seeded in 8‐well confocal dishes and cultured for 24 h. After incubated with 0.25 mm MPP^+^ for 24 h to mimic an inflammatory environment, the cells were incubated with CSCC‐FITC for 2 h, followed by addition of the MitoTracker Red CMXRos working solution (Cat. no. C1049B, Beyotime) and culturing for 30 min at 37 °C. After washing three times with fresh medium, the fluorescence of cells were observed with a CLSM (FV1200, Olympus, Japan, excitation: 549 nm, 488 nm, emission: 599 nm and 516 nm, respectively).

### Immunofluorescence Staining

SH‐SY5Y cells were seeded in the circle microscope cover glass (Nest) with a density of 1 × 10^4^–2 × 10^4^ cells per well in 6‐well plates. After the cells were attached to the glass firmly, they were pretreated with CSC NPs (6.25, 12.5, 25 µm), Cur (25 µm), CSCCT NPs (25 µm) for 2 h and EX527 inhibitor (10 µm) for 12 h, and stimulated with 3 mm MPP^+^ for another 24 h. The cells were washed with PBS twice and fixed with 4% paraformaldehyde for 30 min at 4 °C. Then, the cells were exposed to 0.2% Triton solution (Cat. no. t109026, Aladdin) for 30 min at room temperature, and washed with PBS for three times (3 min each time). After the cells were blocked with 5% bovine serum albumin (Sinopharmatic Chemical Reagent Suzhou Co., Ltd.) at 37 °C for 1 h, they were incubated with the corresponding diluted antibody overnight in the dark at 4 °C. The primary antibodies were diluted as follows: anti‐SIRT1 (1:200, Cat. no. ab189494, abcam), anti‐PGC‐1*α* (1:200, Cat. no. 66369, Prointech), anti‐Ndufs1 (1:200, Cat. no. ab169540, abcam), anti‐Cytochrome c (1:200, Cat. no. 11940, Cell Signaling Technology), anti‐TFAM (1:200, Cat. no. AF8127, Beyotime), and anti‐cleaved caspase‐3 (Asp175) (1:400, Cat. no. 9664, Cell Signaling Technology). Then, the cells were washed with PBS three times and incubated with diluted fluorescent dye‐linked secondary antibody for 1 h in the dark at 37 °C, followed by washing with PBS three times again, and immersing in 10 µg mL^−1^ Hoechst 33342 for 10 min at room temperature. The fluorescence images were observed using CLSM (FV1200, Olympus, Japan) after the cells were washed with PBS three times.

### Western Blot

After SH‐SY5Y cells were seeded in 6‐well plates with a density of 1 × 10^4^–2 × 10^4^ cells per well and cultured for 24 h, they were pretreated with CSC NPs (6.25, 12.5, 25 µm), Cur (25 µm), CSCCT NPs (25 µm) for 2 h and SIRT1 inhibitor EX527 (10 µm) for 12 h, and then incubated with 3 mm MPP^+^ for another 24 h. The total proteins were collected by lysing cells with Radio Immunoprecipitation Assay buffer (RIPA, Cat. no. P0013B Beyotime) with protease inhibition cocktails. BCA protein quantification kit (Cat. no. P0010, Beyotime) was used for protein quantification. 30 µg of protein samples were separated by sodium dodecyl sulfate polyacrylamide gel electrophoresis (8% or 12%), and then transferred into polyvinylidene difluoride membranes (0.45 µm, Millipore). The membranes were blocked with 5% nonfat dry milk diluted by 1 × TBST buffer for 2 h at room temperature. Then the membranes were washed with 1 × TBST three times (5 min each time), following by incubation with corresponding specific primary antibodies diluted with QuickBlock Primary Antibody Dilution Buffer (Cat. no. P0256, Beyotime) for overnight at 4 °C. The primary antibodies were diluted as follows: anti‐SIRT1 (1:1000, Cat. no. AF0282, Beyotime), anti‐PGC‐1*α* (1:1000, Cat. no. 2178, Cell Signaling Technology), anti‐Ndufs1 (1:10 000, Cat. no. ab169540, abcam), anti‐Cytochrome c (1:1000, Cat. no. 11940, Cell Signaling Technology), anti‐VCAM‐1 (1:1000, Cat. no. AF1021, Beyotime), anti‐Integrin *α*4 (1:1000, Cat. no. 8440, Cell Signaling Technology), anti‐Integrin *β*1 (1:1000, Cat. no. 9699, Cell Signaling Technology), anti‐*β*‐actin (1:1000, Cat. no. AF5001, Beyotime), and anti‐Lamin B1 (1:1000, ab16048, abcam). After washing with 1 × TBST three times (5 min each time), the membranes were further incubated with corresponding horseradish peroxidase‐conjugated secondary antibodies diluted by 1 × TBST for 1.5 h at room temperature. Then, the membranes were washed three times with 1 × TBST (5 min each time) and visualized by BeyoECL Star reagent (Cat. no. P0018AS, Beyotime), and finally detected by using the FluorChem M System (Protein Simple, San Jose, CA). The quantification of Western blot results was analyzed by ImageJ software.

### The qRT‐PCR Assay for Gene Expression

The qRT‐PCR was applied for determining expression of genes about mitochondrial biogenisis. SH‐SY5Y cells were seeded into 60 mm × 15 mm cell culture dishes with a density of 3 × 10^4^–4 × 10^4^ cells . After being cultured for 24 h, they were pretreated with CSC NPs (6.25, 12.5, and 25 µm), Cur (25 µm), CSCCT NPs (25 µm) for 2 h and SIRT1 inhibitor EX527 (10 µm) for 12 h, respectively, and incubated with fresh medium containing 3 mm MPP^+^ for another 24 h. Then, the cells were collected for qRT‐PCR test and analysis. Intracellular total RNA was extracted with Simply P Total RNA Extraction Kit (Cat. no. BSC52S1, Bioer). After that, 1 µg of RNA was subjected to reverse transcription into cDNA by HiScript II Q RT SuperMix for qPCR (Cat. no. R223‐01, Vazyme Biotech Co., Ltd.). The cDNA was amplificated on ViiA 7 Real‐Time PCR System (ViiA‐7, Life Technologies) and gene expression level was characterized by fluorescence from ChamQ Universal SYBR qPCR Master Mix (Cat. no. Q711‐02‐03, Vazyme Biotech Co., Ltd.). The primer sequences were used as follows: human PGC‐1*α*, forward primer 5′ TCCTCACAGAGACACTAGACA 3′, reverse primer 5′ CTGGTGCCAGTAAGAGCTTCT 3′; human PPAR*γ*, forward primer 5′ TCTGGCCCACCAACTTTGGG 3′, reverse primer 5′ CTTCACAAGCATGAACTCCA 3′; human NRF1, forward primer 5′ AGGAACACGGAGTGACCCAA 3′, reverse primer 5′ TATGCTCGGTGTAAGTAGCCA 3′; human TFAM, forward primer 5′ AGCTCAGAACCCAGATGCAA 3′, reverse primer 5′ CCGCCCTATAAGCATCTTGA 3′; human mitochondrial DNA (COX2), forward primer 5′ GCCAGCCTGACCCATAGCCATAAT 3′, reverse primer 5′ GCCGGCTGCGTATTCTACGTTA 3′; human nuclear 18S, forward primer 5′ ACGGACCAGAGCGAAAGCA 3′, reverse primer 5′ GACATCTAAGGGCATCACAGAC 3′; human CAT, forward primer 5′ TGGAGCTGGTAACCCAGTAGG 3′, reverse primer 5′ CCTTTGCCTTGGAGTATTTGGTA 3′; human GPx4, forward primer 5′ GAGGCAAGACCGAAGTAAACTAC 3′, reverse primer 5′ CCGAACTGGTTACACGGGAA 3′; human SOD2, forward primer 5′ AAGGGAGATGTTACAGCCCAGATA 3′, reverse primer 5′ CCAGAAAATGCTATGATTGATATGAC 3′; human *β*‐actin, forward primer 5′ ACCAACTGGGACGACATGGAGAAA 3′, reverse primer 5′ ATAGCACAGCCTGGATAGCAACG 3′; mouse PGC‐1*α*, forward primer 5′ TTCATCTGAGTATGGAGTCGCT 3′, reverse primer 5′ GGGGGTGAAACCACTTTTGTAA 3′; mouse TFAM, forward primer 5′ GAGCAGCTAACTCCAAGTCAG 3′, reverse primer 5′ GAGCCGAATCATCCTTTGCCT 3′; mouse Gpx4, forward primer 5′ TGTGCATCCCGCGATGATT 3′, reverse primer 5′ CCCTGTACTTATCCAGGCAGA 3′; mouse *β*‐actin, forward primer 5′ GGCTGTATTCCCCTCCATCG 3′, and reverse primer 5′ CCAGTTGGTAACAATGCCATGT 3′. All procedures were carried out according to the manufacturer's instructions. Transcription levels were analyzed by using the Comparative C_T_ (^ΔΔ^C_T_) experiment.

### Characterization of Cell Mitochondrial Morphology by TEM

SH‐SY5Y cells were divided into four groups as mentioned previously: Ctrl group, MPP^+^ group, MPP^+^ and CSC NPs group, and MPP^+^ and CSCCT NPs group. Then, the cells were gently collected from culture flask by cell scrapers, and then fixed with special fixing solution for TEM characterization. Samples were prepared on the nickel or aluminum TEM grids.

### Assessment of the Opening of Blood‐Brain Barrier via Evans Blue

All animals were handled using the protocols approved by the Soochow University Laboratory Animal Center and the University Animal Ethics Committee. The approved certificate number for animal experiments is 220204143.

The BBB was opened and evaluated through the staining with EB, according to the previously reported method with a slight improvement.^[^ ^63^ ^]^ A US transducer (1 MHz and 37 mm diameter) was used to open the BBB of mice temporarily, which was driven by a function generator connected to a power amplifier. A movable cone filled with degassed water was employed to hold the transducer in place and direct the US beam into the brain. The acoustic parameters were set as follows: 25 amp, 1.0 MHz frequency, 1 ms pulse interval, and 90 s sonication duration. 50 µL of MB with a mean diameter of ≈2 µm and concentration of ≈1 × 10^9^ bubbles mL^−1^ were injected into mice via the tail vein before sonication. To confirm the successful opening and recovery of the BBB, the mice were intravenously injected with 2% EB dye, and then sacrificed at 2 h after EB injection. The brain tissue was collected and taken pictures for use.

### Assessment of Targeting Capability of CSCCT NPs by PA Imaging and Staining Slices of Brain In Vivo

The nude mice were monitored with a Multispectral optoacoustic tomography scanner (MSOT, iThera Medical) for 8 h, after they were sonicated to open their BBB and then intravenously injected with CSCCT NPs (dose: 5 mg kg^−1^). The PA images of the mice were captured at every hour after injection to analyze and assess the in vivo targeting capability of CSCCT NPs with the assistance of US.

The healthy and MPTP‐induced PD mice were divided into three groups, i.e., I) healthy mice injected with CSCCT‐FITC NPs through their tail veins after US sonication (Ctrl + US + CSCCT‐FITC group), II) MPTP‐induced PD mice injected with CSCC‐FITC NPs through their tail veins after US sonication (PD + US + CSCC‐FITC group), and III) MPTP‐induced PD mice injected with CSCCT‐FITC NPs through their tail veins after US sonication (PD + US + CSCCT‐FITC group) (dose: 5 mg kg^−1^). The TH in their brain tissue slices was stained by immunofluorescence assay to evaluate the ability of CSCCT NPs to target neurons at the inflammatory site.

### Treatment of MPTP‐Induced Parkinson's Disease

14–16 weeks old male C57BL/6J mice were used for all experiments. PD mice were induced by repeatedly intraperitoneal injections of MPTP solution for 8 days (30 mg kg^−1^).^[^ ^38^ ^]^ All the mice were randomly divided into the following five groups (*n* = 10): 1) Ctrl group (healthy and wild type mice), 2) PD group (PD mice without any treatment), 3) PD + NS + US group (PD mice were injected with normal saline solution after US sonication), 4) PD + CSCCT group (PD mice were injected with CSCCT NPs), and 5) PD + US + CSCCT group (PD mice were injected with CSCCT NPs and US sonication). These mice were received twice treatments to ensure therapeutic efficacy.

### Rotary Rod Test

The fatigue and the grip strength of the mice were observed by rotary rod test. The speed and time of rotary rod were 30 rpm and 4 min, respectively. The time of the mice spent on the rod was recorded by small animal rotating rod fatigue instrument (XR‐YLS‐4C, Shanghai Xinruan Information Technology Co., Ltd).

### Morris Water Maze Test

Morris water maze was used to assess the spatial learning and memory performance of mice, which were trained and tested in a water maze with a diameter of 1200 mm. The water temperature need to be maintained at 20 ± 2 °C and the water was 1 cm above the platform, which was invisible to mice. The water maze was divided into four quadrants and marked with homologous four equidistant points. The mice were trained four times a day for four days and tested on the fifth day. Specifically, the mice traveled from four different quadrants and had 1 min to find the platform during the training. The trigger event was set to stop recording tracks if the mice stay on the platform for more than 3 s. Regardless of whether the trigger event was triggered or not, the mice were guided to the platform and stayed for 10 s. At the end of training, and the platform was removed on the fifth day. The SuperMaze Morris Video analysis system (XR‐XM101, Shanghai Xinruan Information Technology Co., Ltd) was applied for recording and analyzing the trajectory of each mouse. The residence time of the mouse in the target quadrant and the frequency of crossing the target quadrant and the times of crossing the original platform were recorded.

### Open Field Test

The open field test is performed to assess the locomotory and exploratory behavior of mice. The size of apparatus was 50 × 50 × 40 cm. Each mouse was placed in one designated corner, and its activities were recorded during the subsequent 10 min and then assessed by a SuperMaze Morris Video analysis system (XR‐XZ301, Shanghai Xinruan Information Technology Co., Ltd). Zone transition number and mean speed in zone‐total were calculated and analyzed.

### Elevated Plus‐Maze Test

Elevated plus‐maze test is based on the contradiction between the fear of mouse in an open arm environment and its ability to explore a new environment to examine anxiety state. The size of open and close arm was 65 cm, each mouse was placed in the center zone with the head toward the close arm, and the movement path of the mouse was recorded for 5 min, then assessed by a SuperMaze Morris Video analysis system (XR‐XG201, Shanghai Xinruan Information Technology Co., Ltd). The open arm entries and the time of open arm were calculated and analyzed.

### H&E Staining

The major organs (i.e., heart, liver, spleen, lung, kidney) of five groups of mice were stained with H&E for investigating the biocompatibility of CSCCT NPs. All above staining procedures were carried out by Servicebio Biotechnology Co., Ltd., Wuhan.

### Statistical Analysis

All data represented as mean ± SD using GraphPad Prism 8.0, the sample size (*n*) for statistical analysis was shown in the figure captions. The original data without any preprocessing passed the normality and homogeneity of variance were used for the subsequent analysis. The testing level alpha was set at 0.05 for the following two statistical methods. The two‐sided unpaired Student's *t*‐test and the two‐sided one‐way ANOVA with a Tukey post hoc analysis were used for comparisons between two groups, and among multiple groups, respectively. The data were classified by the values of *P*. *P < 0.05, **P < 0.01, ***P < 0.001, ****P < 0.0001, ns, not significant.

## Conflict of Interest

The authors declare no conflict of interest.

## Author Contributions

Q.Z.: data curation, investigation, and writing the original draft; H.L.: data curation, investigation, and methodology; H.Z.: data curation, investigation, and methodology; Y.H.: data curation, investigation, and methodology; J.Y.: data curation, investigation, and methodology; T.W.: data curation, investigation, and methodology; Y.G.: data curation, investigation, and methodology; Z.L.: conceptualization, data curation, funding acquisition, project administration, supervision, and writing, reviewing, and editing.

## Supporting information

Supporting Information

## Data Availability

The data that support the findings of this study are available from the corresponding author upon reasonable request.
