# Supplementary material for: Ameliorating Mitochondrial Dysfunction of Neurons by Biomimetic Targeting Nanoparticles Mediated Mitochondrial Biogenesis to Boost the Therapy of Parkinson's Disease
Source: Adv Sci (Weinh). 2023 May 18;10(22):2300758. doi: 10.1002/advs.202300758 (PMC10401119; doi:10.1002/advs.202300758)
Supplement: Supplementary file 1 — Supporting Information [file ADVS-10-2300758-s001.pdf]

ADVANCED SCIENCE
Open Access

Supporting Information

for *Adv. Sci.*, DOI 10.1002/advs.202300758

Ameliorating Mitochondrial Dysfunction of Neurons by Biomimetic Targeting Nanoparticles Mediated Mitochondrial Biogenesis to Boost the Therapy of Parkinson's Disease

*Qing Zheng, Hanghang Liu, Hao Zhang, Yaobao Han, Jiaxin Yuan, Tingting Wang, Yifan Gao and Zhen Li**

Supporting information

Ameliorating Mitochondrial Dysfunction of Neurons by Biomimetic Targeting Nanoparticles Mediated Mitochondrial Biogenesis to Boost the Therapy of Parkinson's Disease

Qing Zheng, Hanghang Liu, Hao Zhang, Yaobao Han, Jiaxin Yuan, Tingting Wang, Yifan Gao, Zhen Li*

Center for Molecular Imaging and Nuclear Medicine, State Key Laboratory of Radiation Medicine and Protection, School for Radiological and Interdisciplinary Sciences (RAD-X), Suzhou Medical College of Soochow University, Collaborative Innovation Center of Radiation Medicine of Jiangsu Higher Education Institutions Suzhou 215123, China

Email: zhenli@suda.edu.cn

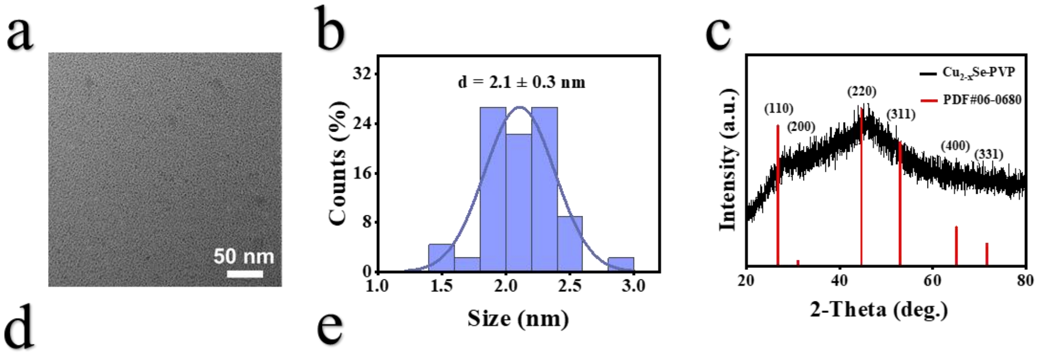

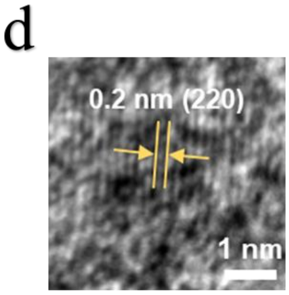

e

Element	Atomic Fraction (%)	Mass Fraction (%)
Cu	33.87	49.61
Se	19.27	34.30
N	26.85	8.70
O	20.00	7.40

Figure S1. a-b) TEM image and particle size distribution of CSP NPs. c) XRD pattern of CSP NPs in comparison with the standard diffraction peaks of cubic berzelianite (JCPDS Card No. 06–0680). d-e) Corresponding high-resolution transmission electron microscopy (HRTEM) and energy dispersive spectrometer (EDS) analysis of CSP NPs.

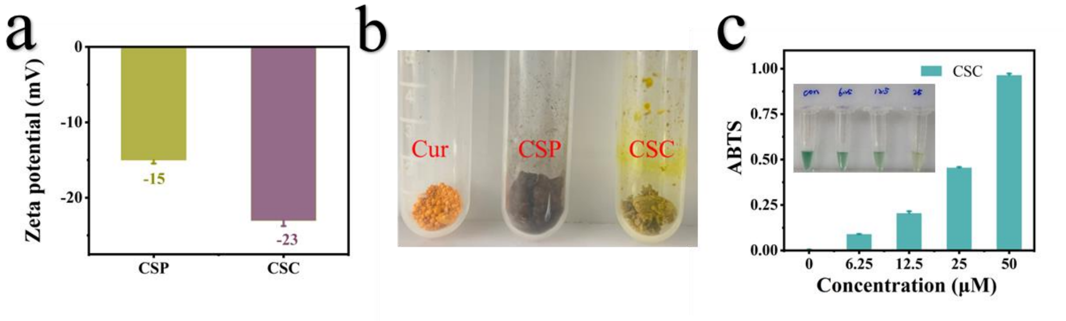

Figure S2. a) Zeta potential of CSP NPs and CSC NPs. b) Digital picture of Cur, CSP NPs and CSC NPs powders. c) Total antioxidative capability of CSC NPs with the corresponding photo of solutions (n = 3).

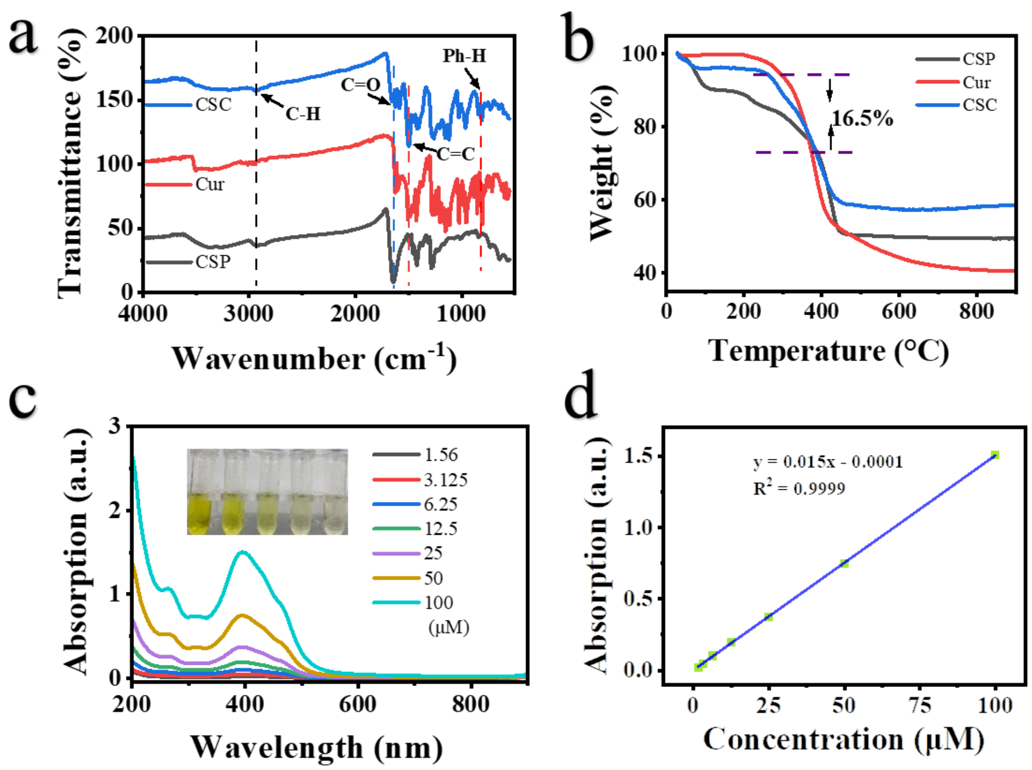

Figure S3. a) Fourier-transform infrared spectroscopy (FTIR) spectra of Cur, CSP NPs, and CSC NPs. b) Thermogravimetric analysis (TGA) curves of Cur, CSP NPs, and CSC NPs. c) UV-*vis* absorption of various concentrations of CSC NPs (inset is the picture of CSC NPs solutions with a concentration of 3.125, 6.25, 12.5, 25 and 50 μM respectively from right to left). d) The fitted linear relationship of CSC NPs absorbance and their concentration.

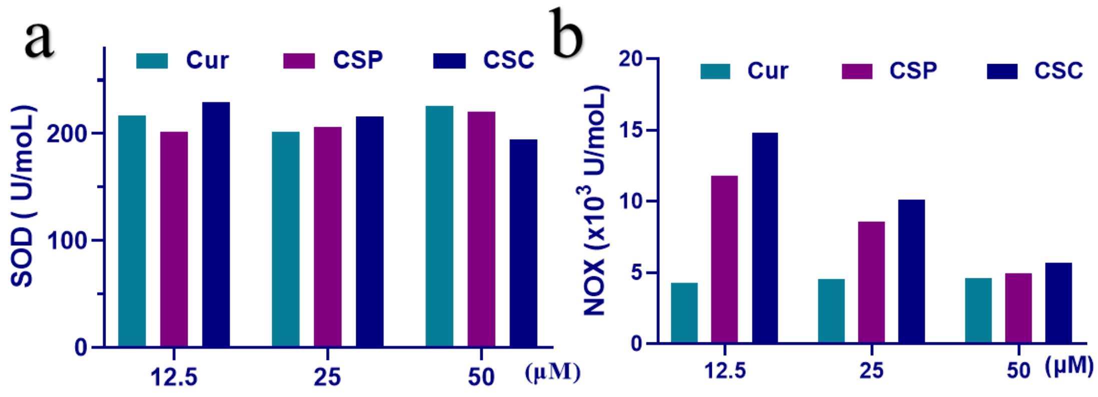

Figure S4. Comparison of a) the SOD-like activity and b) the NOX-like activity of Cur, CSP NPs.

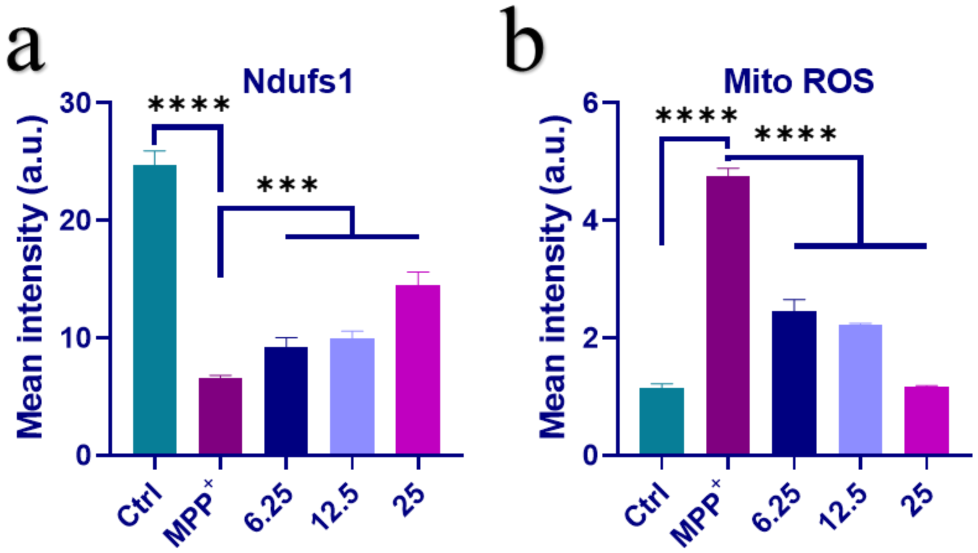

Figure S5. Quantitative analysis of mean fluorescence intensity from a) Ndufs1 and b) Mito ROS (n = 3). The two-sided one-way ANOVA with a Tukey post-hoc analysis was used for comparison among multiple groups. *P < 0.05, **P < 0.01, ***P < 0.001, ****P < 0.0001, ns, not significant.

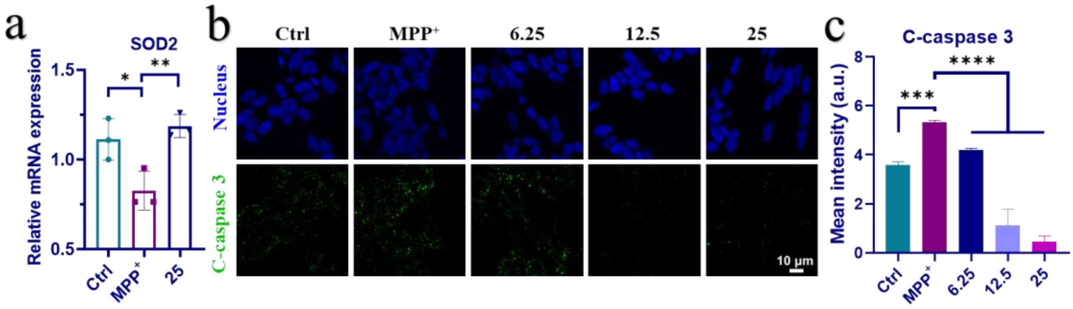

Figure S6. a) Gene expression of antioxidative SOD2 enzyme analyzed by qRT-PCR. b) CLSM images indicated the expression of cleaved-caspase 3 in SH-SY5Y cells pretreated by different concentration CSC NPs incubated with MPP^+. c) Quantitative analysis of fluorescence intensity of C-caspase 3. Blue: cell nuclei; green: C-caspase 3 (n = 3). The two-sided one-way ANOVA with a Tukey post-hoc analysis was used for comparison among multiple groups. **P* < 0.05, ***P* < 0.01, ****P* < 0.001, *****P* < 0.0001, ns, not significant.

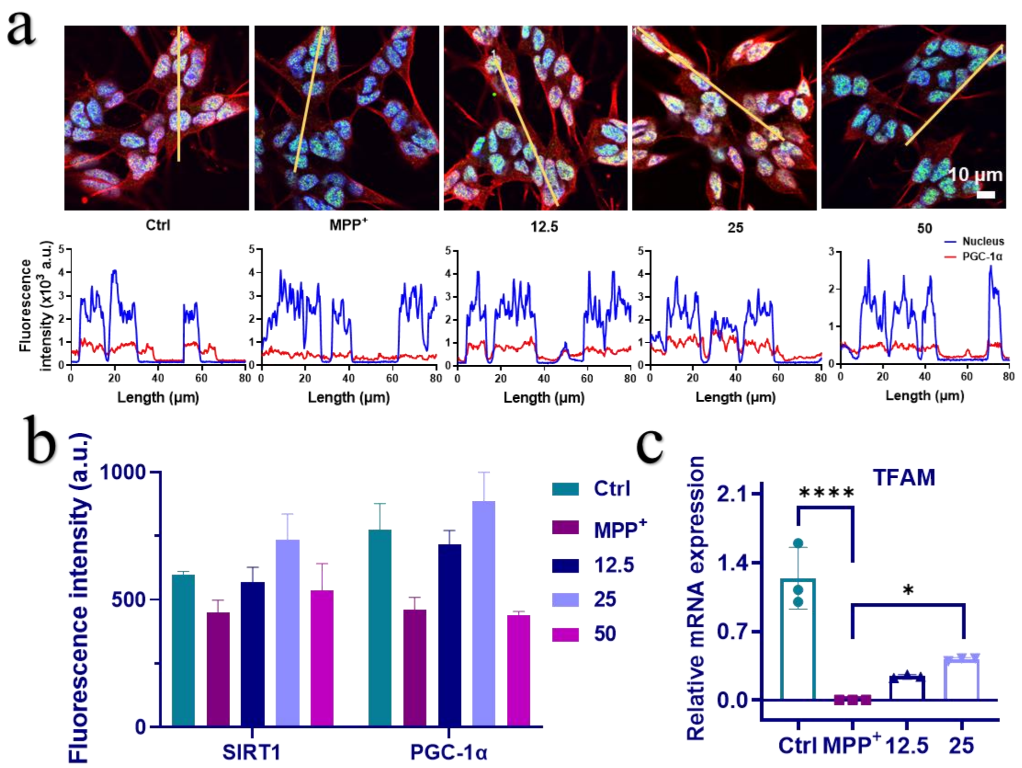

Figure S7. a) Co-location analysis of PGC-1α and nuclei. b) Quantitative analysis of fluorescence intensity from SIRT1 and PGC-1α in SH-SY5Y cells after the cells were incubated with different concentrations of CSC NPs. c) Gene expression of mitochondrial biogenesis-related TFAM analyzed by qRT-PCR (n = 3). The two-sided one-way ANOVA with a Tukey post-hoc analysis was used for comparison among multiple groups. *$P < 0.05$, **$P < 0.01$, ***$P < 0.001$, ****$P < 0.0001$, ns, not significant.

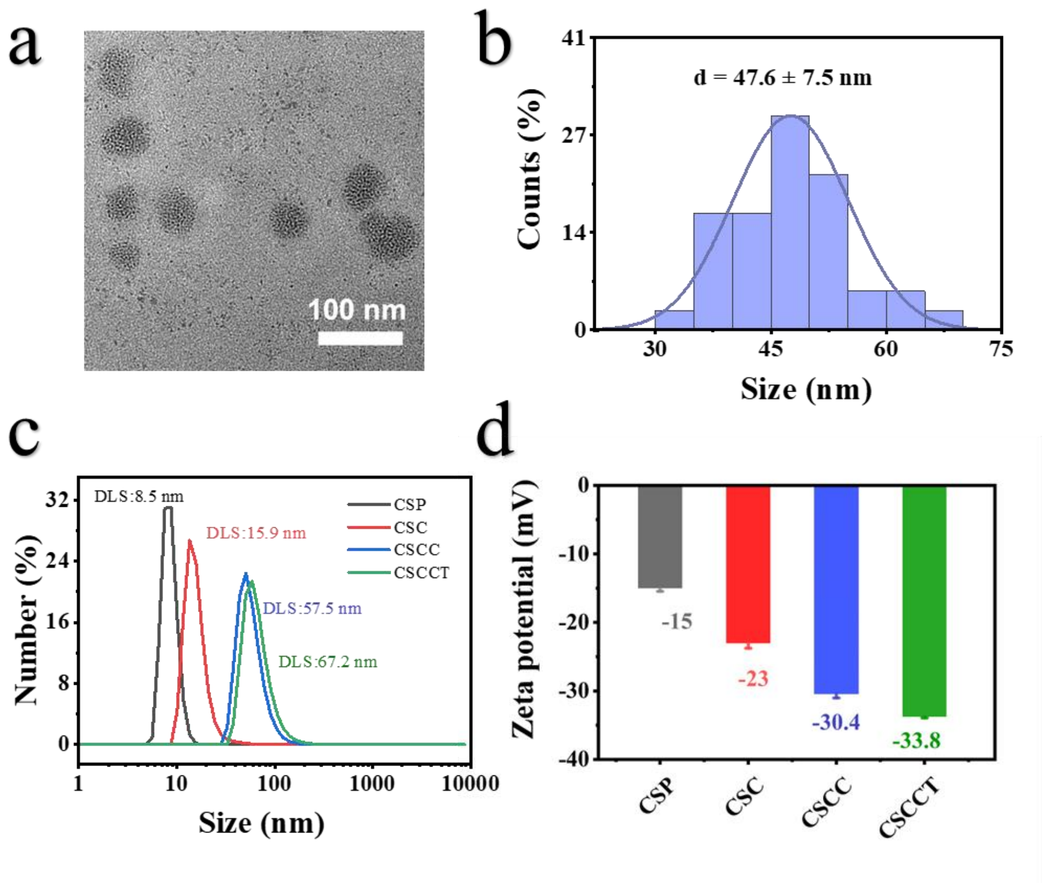

Figure S8. a-b) TEM image and particle size distribution of CSCCT NPs. c) Hydrodynamic particle size and d) zeta potential of CSP NPs, CSC NPs, CSCC NPs and CSCCT NPs (n = 3).

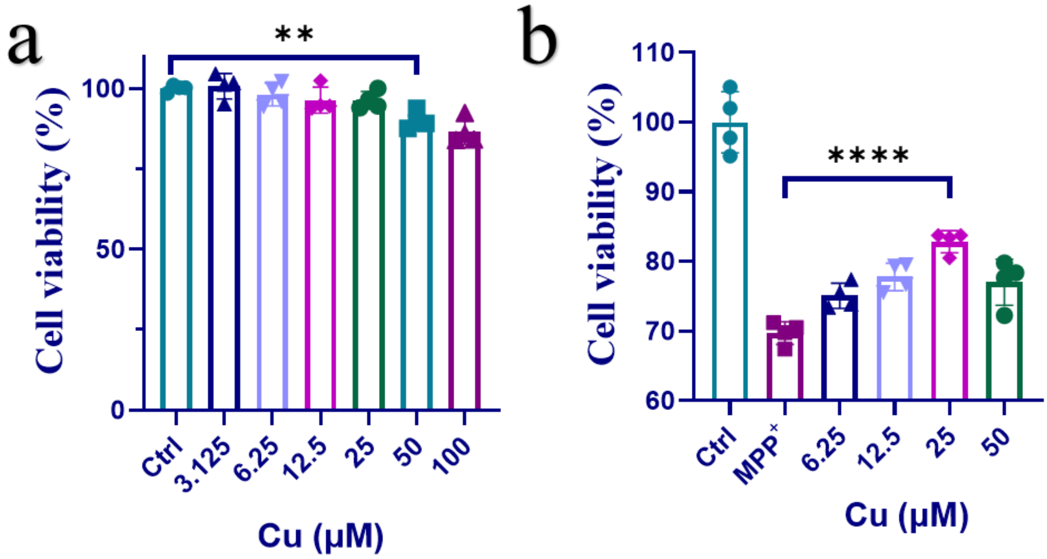

Figure S9. a) Cell viability of SH-SY5Y cells after treatment with different concentrations of CSCCT NPs for 2 h (n = 4). b) Cell viability of SH-SY5Y cells after pre-treatment with different concentrations of CSCCT NPs, and then treatment with 3 mM MPP^+ (n = 4). The two-sided one-way ANOVA with a Tukey post-hoc analysis was used for comparison among multiple groups. *$P < 0.05$, **$P < 0.01$, ***$P < 0.001$, ****$P < 0.0001$, ns, not significant.

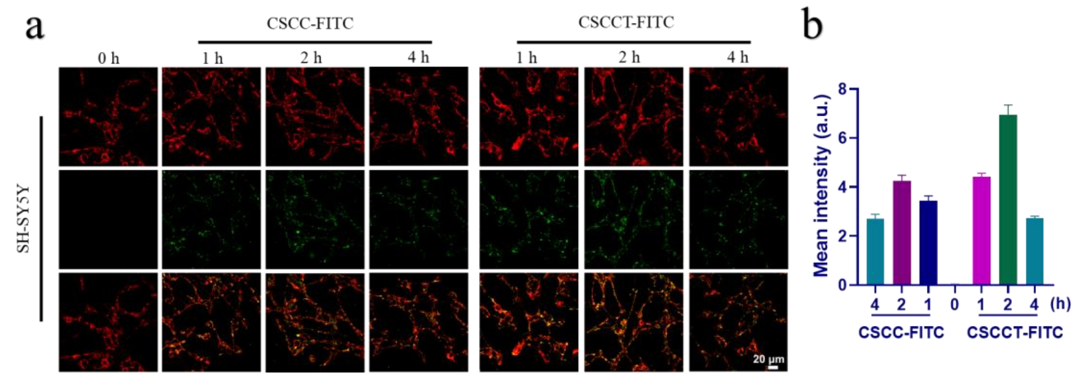

Figure S10. a) CLSM images of mitochondria in SH-SY5Y cells after incubation with FITC-labeled nanoparticles (CSCC-FITC NPs and CSCCT-FITC NPs, 25 μM) for 1 h, 2 h and 4 h, respectively. Blue: cell nuclei; green: FITC-labeled nanoparticles; red: mitochondria. b) Quantitative analysis of FITC-labeled nanoparticles (n = 3).

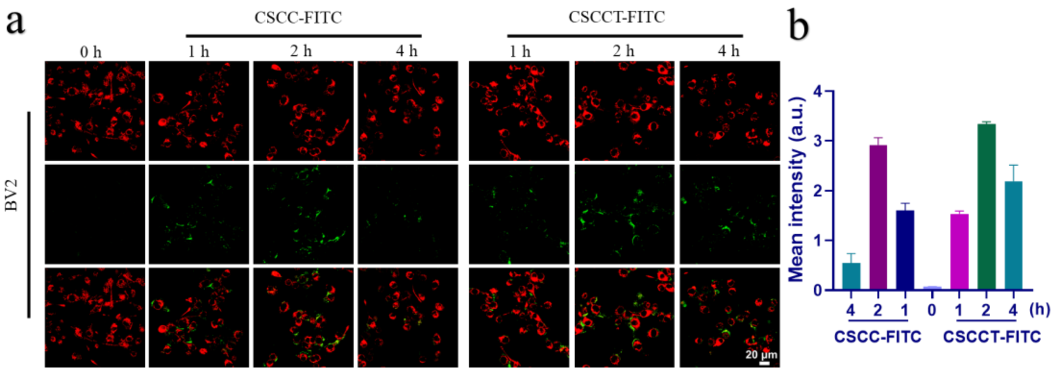

Figure S11. a) CLSM images of mitochondria in BV2 cells after incubation with FITC-labeled nanoparticles (CSCC-FITC NPs and CSCCT-FITC NPs, 25 μM) for 1 h, 2 h and 4 h, respectively. Blue: cell nuclei; green: FITC-labeled nanoparticles; red: mitochondria. b) Quantitative analysis of FITC-labeled nanoparticles (n = 3).

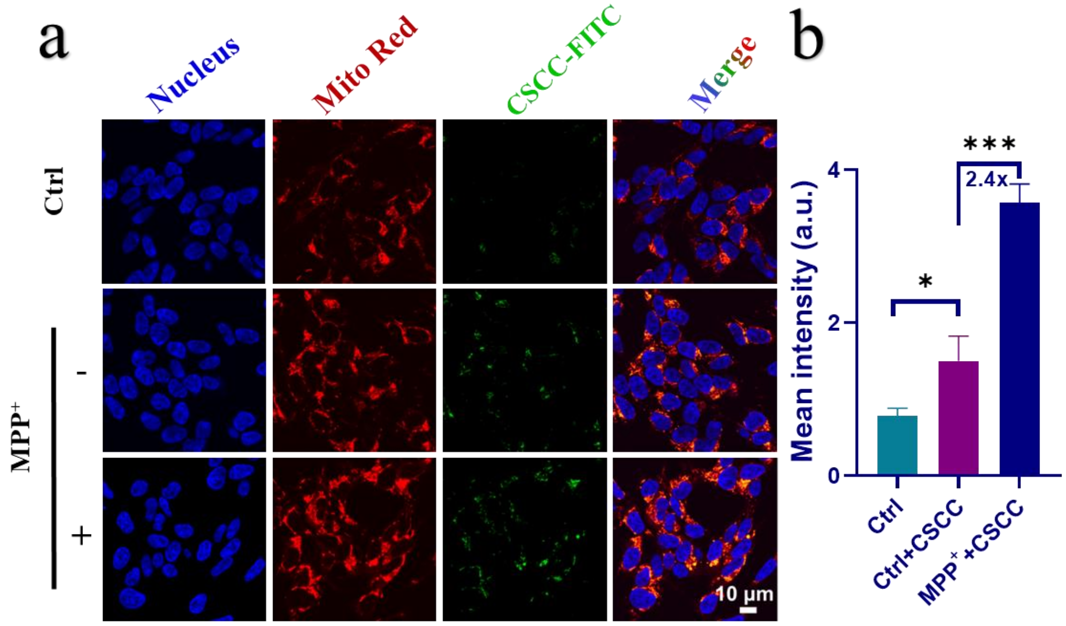

Figure S12. a) CLSM images of mitochondria in SH-SY5Y cells after the cells were treated with or without 0.25 mM MPP^+ for 24 h to mimic an inflammatory environment, followed by incubation with FITC labeled CSCC NPs. Blue: cell nuclei; green: FITC-labeled nanoparticles; red: mitochondria. b) Quantitative analysis of FITC-labeled nanoparticles (n = 3). The two-sided one-way ANOVA with a Tukey post-hoc analysis was used for comparison among multiple groups. *$P < 0.05$, **$P < 0.01$, ***$P < 0.001$, ****$P < 0.0001$, ns, not significant.

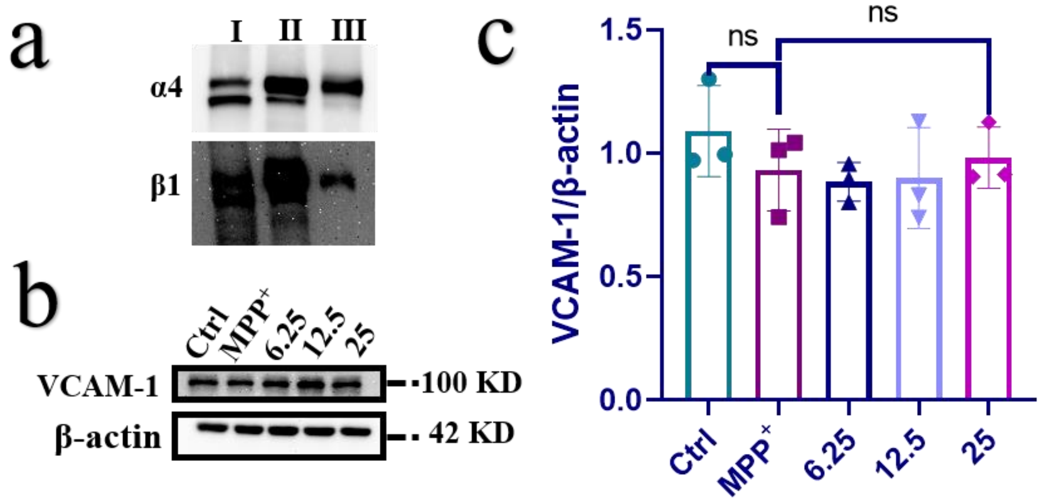

Figure S13. a) Western blot analysis of specific proteins on the membrane, including integrin α4 and β1 proteins from (I) RAW 264.7 cell lysates, (II) RAW 264.7 cell membrane, and (III) CSCCT NPs. b-c) Western blot analysis of VCAM-1 protein from SH-SY5Y cells incubated with MPP^+ and different concentrations of CSCCT NPs (n = 3). The two-sided one-way ANOVA with a Tukey post-hoc analysis was used for comparison among multiple groups. *$P < 0.05$, **$P < 0.01$, ***$P < 0.001$, ****$P < 0.0001$, ns, not significant.

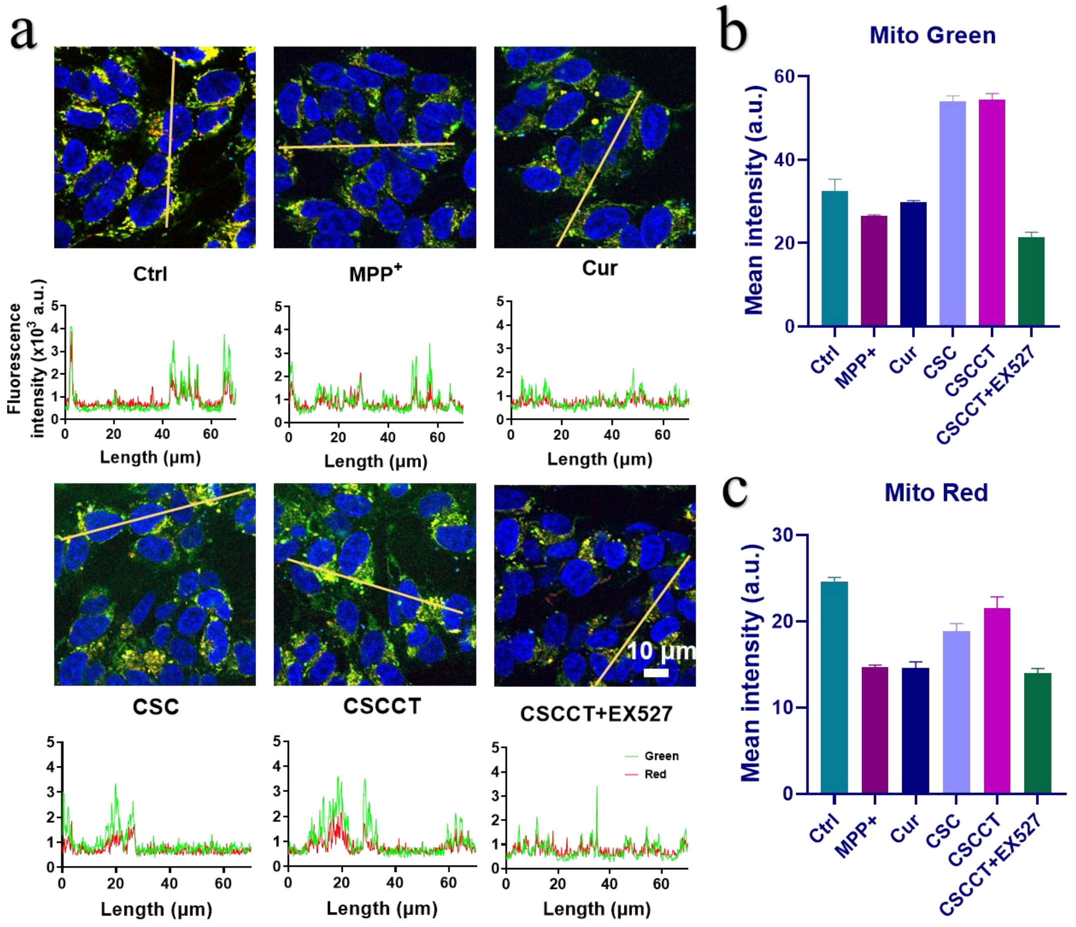

Figure S14. a) CLSM images and co-location analysis of total and active mitochondria in SH-SY5Y cells after different treatment. b-c) Quantitative analysis of green and red fluorescence. Blue: nuclei; green: total mitochondria; red: active mitochondria (n = 3).

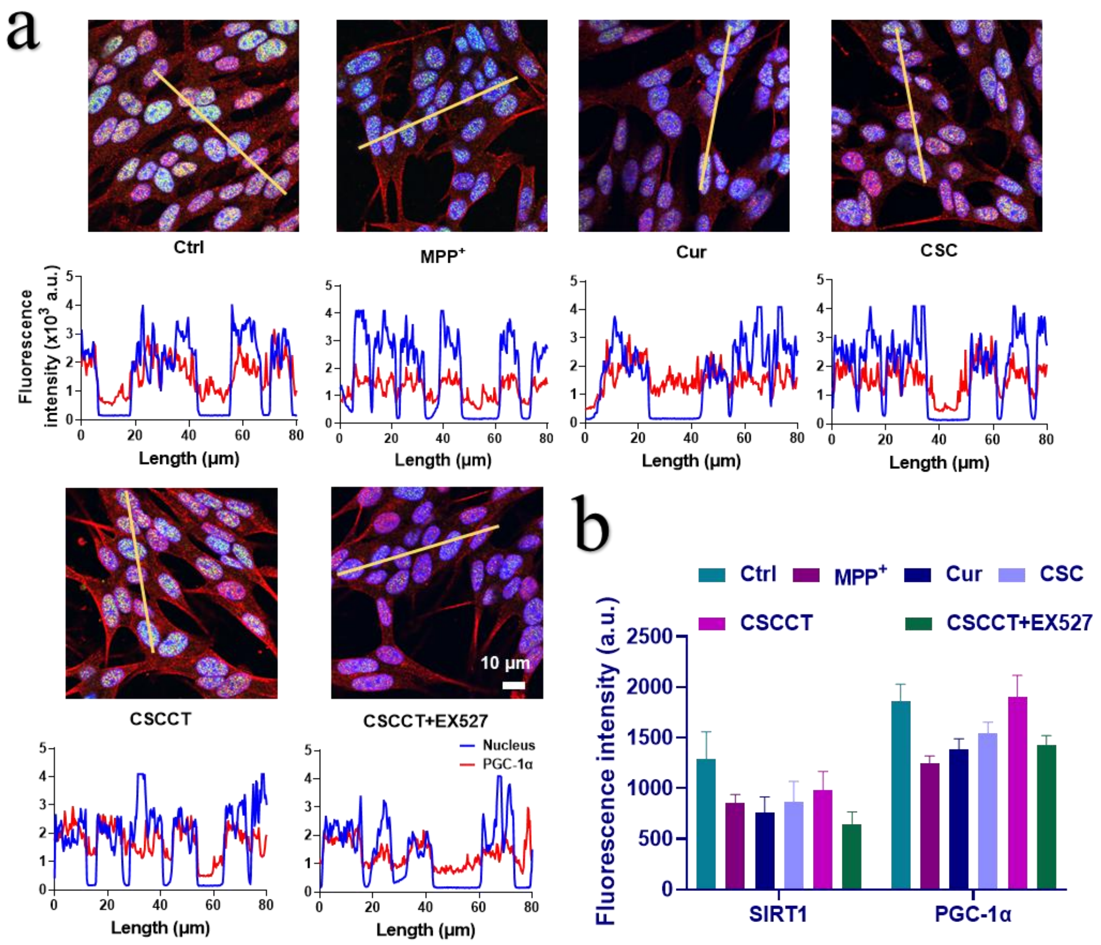

Figure S15. (a) CLSM images and co-location analysis of PGC-1α and nucleus in SH-SY5Y cells after different treatment. (b) Quantitative analysis of green and red fluorescence. Blue: nuclei; green: SIRT1; red: PGC-1α (n = 3).

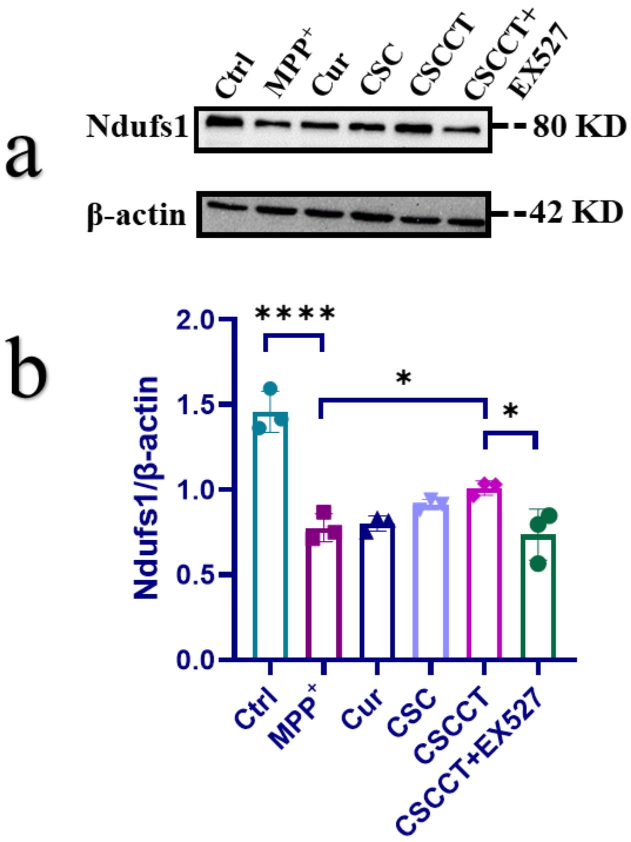

Figure S16. a) Western blot and b) relative quantitative analysis of Ndufs1 protein in SH-SY5Y cells after treatment with Cur (25 μM), CSC NPs (25 μM), CSCCT NPs (25 μM), CSCCT NPs (25 μM) and EX527 (10 μM) (n = 3). The two-sided one-way ANOVA with a Tukey post-hoc analysis was used for comparison among multiple groups. *$P < 0.05$, **$P < 0.01$, ***$P < 0.001$, ****$P < 0.0001$, ns, not significant.

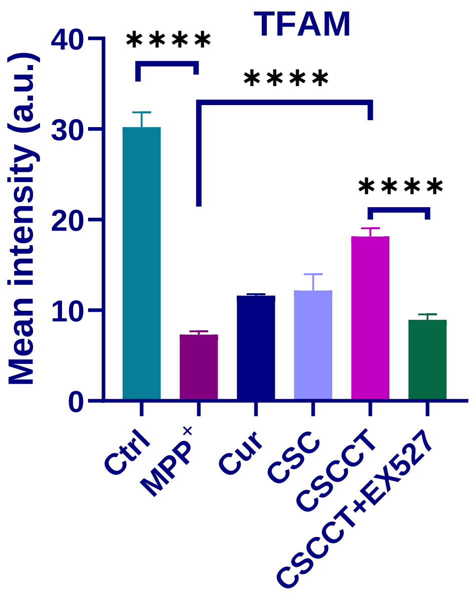

Figure S17. Quantitative analysis of fluorescence intensity from TFAM in SH-SY5Y cells after the cells were pretreated with Cur (25 μM), CSC NPs (25 μM), and CSCCT NPs (25 μM) for 2 h, followed by incubation with 3 mM MPP^+ for 24 h (n = 3). The two-sided one-way ANOVA with a Tukey post-hoc analysis was used for comparison among multiple groups. *$P < 0.05$, **$P < 0.01$, ***$P < 0.001$, ****$P < 0.0001$, ns, not significant.

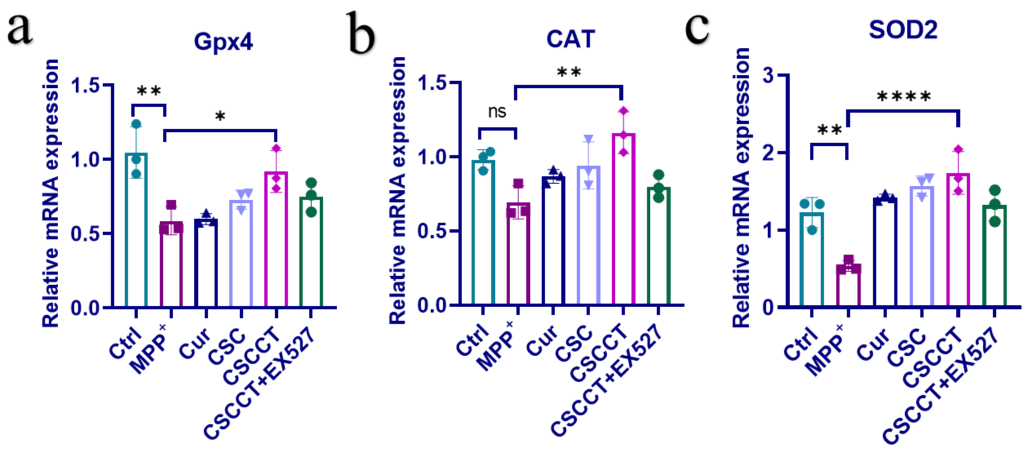

Figure S18. Gene expressions of a) Gpx4, b) CAT, and c) SOD2 in SH-SY5Y cells after treatment with Cur (25 μM), CSC NPs (25 μM), CSCCT NPs (25 μM), CSCCT NPs (25 μM) and EX527 (10 μM), analyzed by qRT-PCR (n = 3). The two-sided one-way ANOVA with a Tukey post-hoc analysis was used for comparison among multiple groups. *$P < 0.05$, **$P < 0.01$, ***$P < 0.001$, ****$P < 0.0001$, ns, not significant.

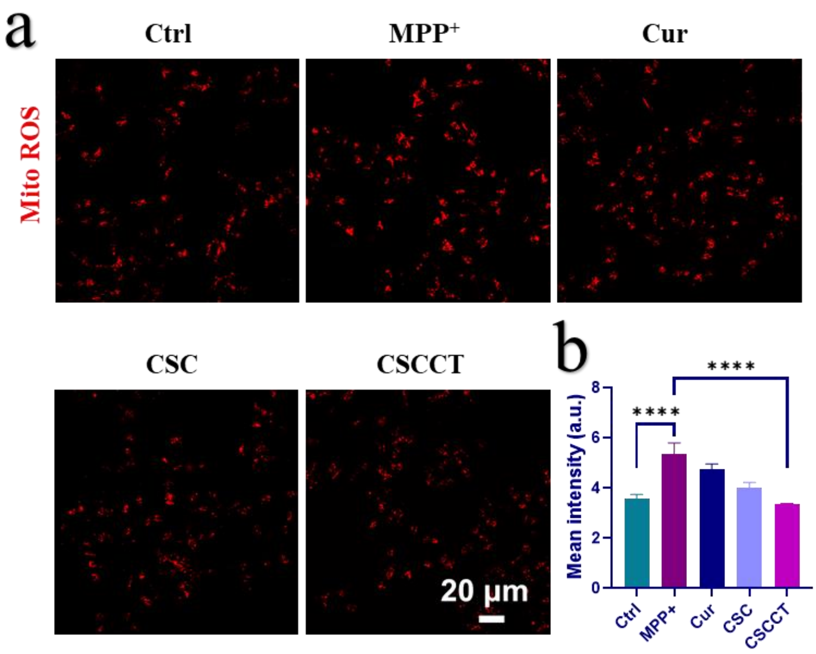

Figure S19. a) CLSM images and b) quantitative analysis of mitochondrial ROS generated in SH-SY5Y cells (red fluorescence) after the cells were pretreated with Cur (25 μM), CSC NPs (25 μM), and CSCCT NPs (25 μM) for 2 h, followed by incubation with 3 mM MPP^+ for 24 h (n = 3). The two-sided one-way ANOVA with a Tukey post-hoc analysis was used for comparison among multiple groups. *$P < 0.05$, **$P < 0.01$, ***$P < 0.001$, ****$P < 0.0001$, ns, not significant.

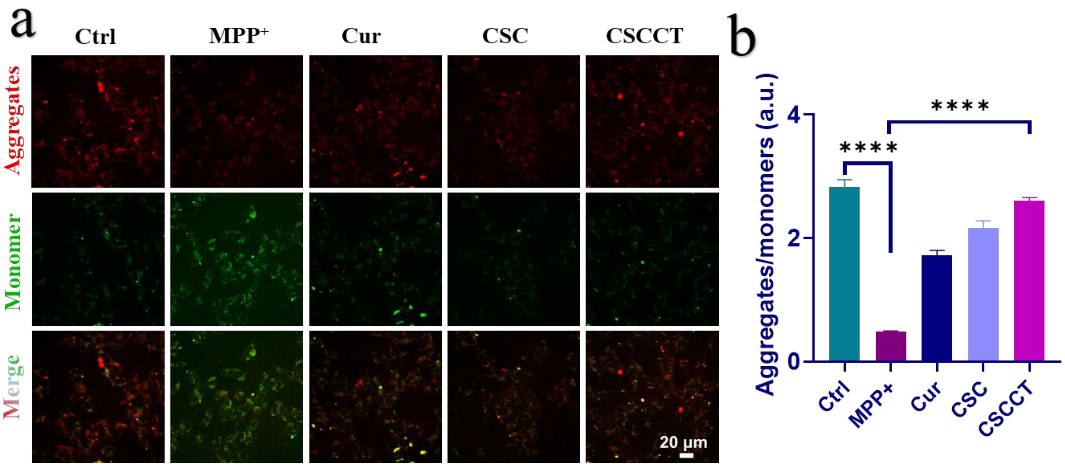

Figure S20. a) CLSM images and b) quantitative analysis of mitochondrial membrane potential (MMP) of SH-SY5Y cells after the cells were pretreated with Cur (25 μM), CSC NPs (25 μM), and CSCCT NPs (25 μM) for 2 h, followed by incubation with MPP^+. Red: aggregates; green: monomer (n = 3). The two-sided one-way ANOVA with a Tukey post-hoc analysis was used for comparison among multiple groups. *$P < 0.05$, **$P < 0.01$, ***$P < 0.001$, ****$P < 0.0001$, ns, not significant.

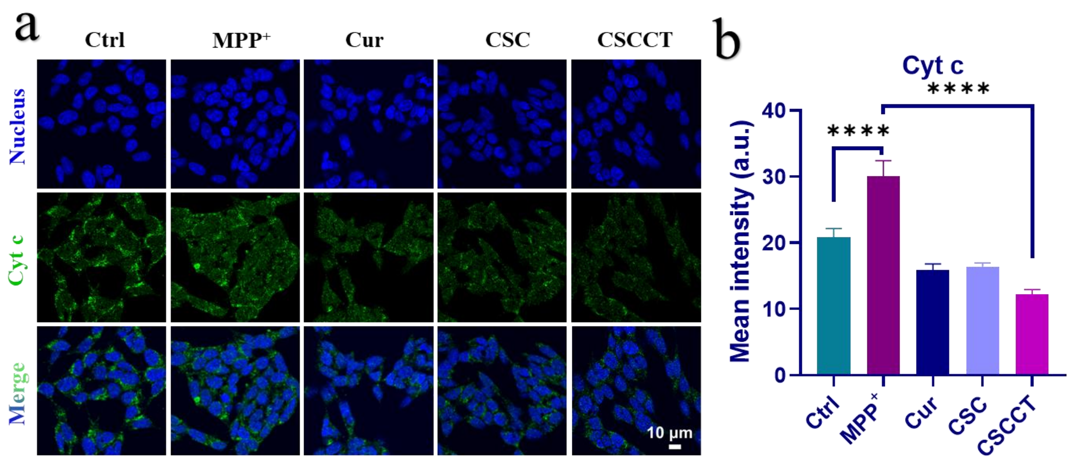

Figure S21. a) CLSM images and b) quantitative analysis of the cytochrome c (Cyt c) of SH-SY5Y cells after the cells were pretreated with Cur (25 μM), CSC NPs (25 μM), CSCCT NPs (25 μM) for 2 h, followed by incubation with MPP^+. Blue: nuclei; green: Cyt c (n = 3). The two-sided one-way ANOVA with a Tukey post-hoc analysis was used for comparison among multiple groups. **P* < 0.05, ***P* < 0.01, ****P* < 0.001, *****P* < 0.0001, ns, not significant.

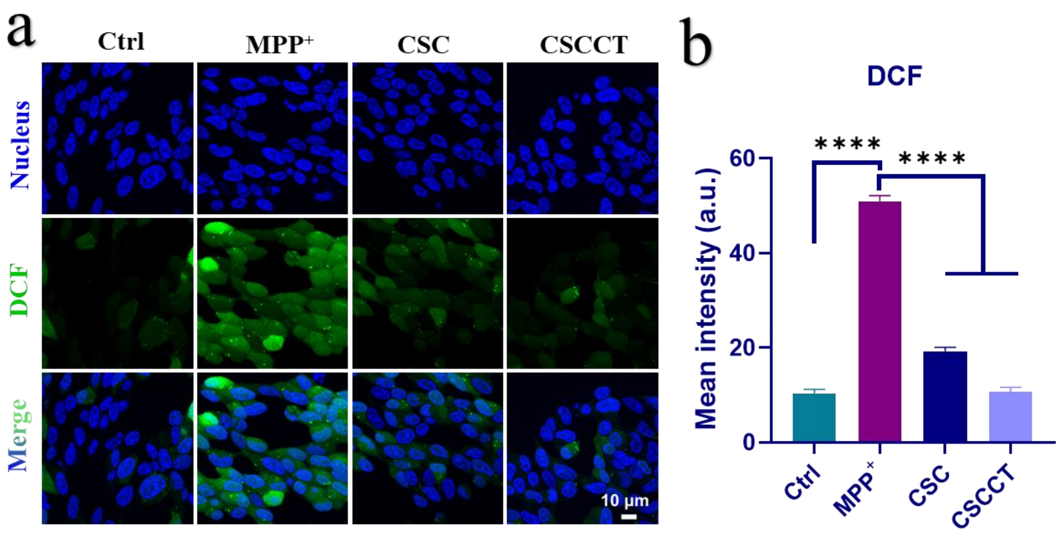

Figure S22. a) CLSM images and b) quantitative analysis of fluorescence intensity of DCF in SH-SY5Y cells for the detection of intracellular total ROS, before and after they were pretreated with CSC NPs and CSCCT NPs and then incubated with MPP^+. Blue: cell nuclei; green: DCF fluorescence generated from the oxidization of non-fluorescent DCFH by ROS (n = 3). The two-sided one-way ANOVA with a Tukey post-hoc analysis was used for comparison among multiple groups. **P* < 0.05, ***P* < 0.01, ****P* < 0.001, *****P* < 0.0001, ns, not significant.

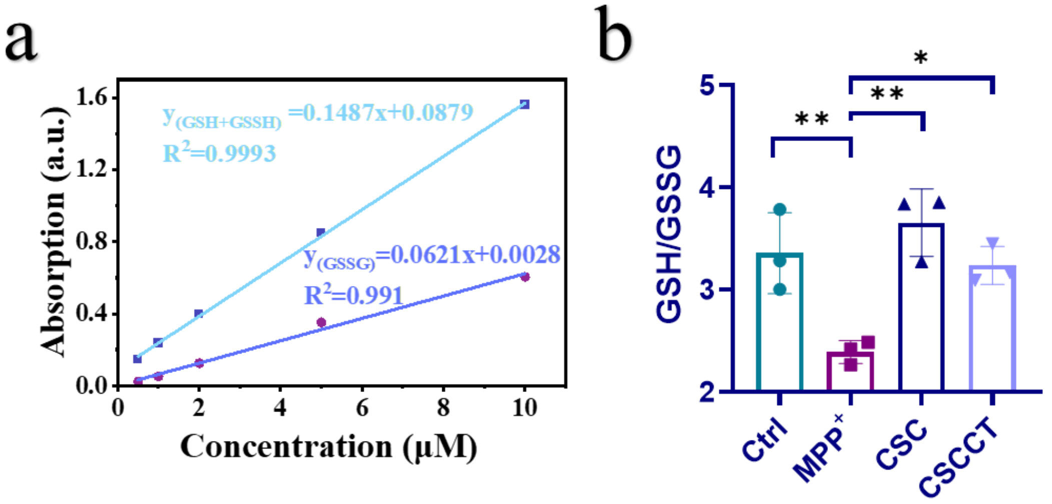

Figure S23. a) The absorption linear dependence of reduced and total glutathione (GSH) on their concentrations. b) The ratio of GSH to GSSG in non-treated SH-SY5Y cells and the cells pretreated with CSC NPs and CSCCT NPs (n = 3). The two-sided one-way ANOVA with a Tukey post-hoc analysis was used for comparison among multiple groups. *$P < 0.05$, **$P < 0.01$, ***$P < 0.001$, ****$P < 0.0001$, ns, not significant.

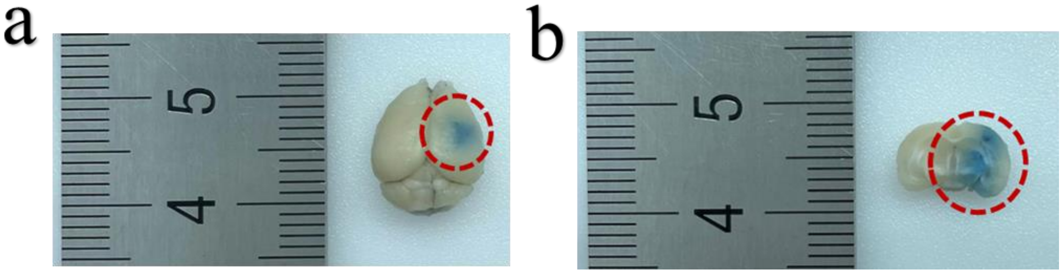

Figure S24. a) The whole and b) the coronal plane of the brain dissected from nude mouse. The staining of EB indicated the successful opening of BBB by US (n = 3).

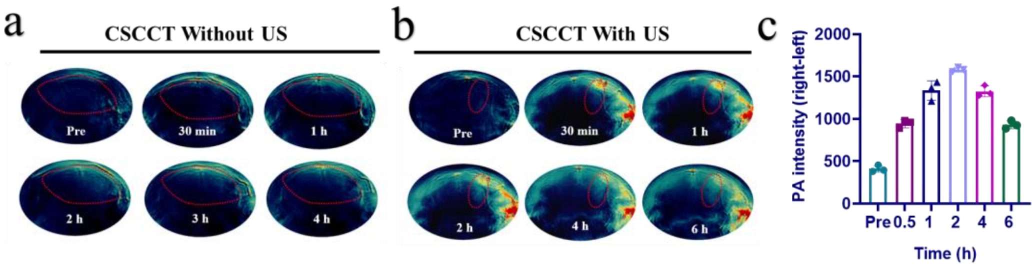

Figure S25. a-b) Photoacoustic imaging (PA) of brain collected from nude mice, of which their BBB was opened or not opened with US, followed by intravenous injection of CSCCT NPs. c) PA signal intensity of brain from the nude mice treated with CSCCT NPs and US (n = 3).

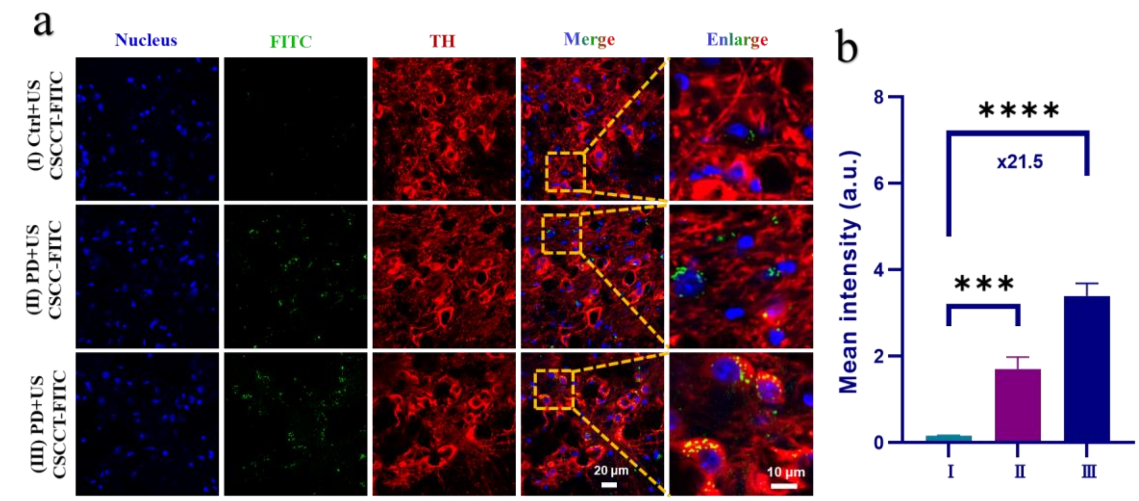

Figure S26. a) CLSM images and b) corresponding quantitative analysis of fluorescence intensity from FITC-labeled nanoparticles in the substantia nigra of right brain slices from (I) Ctrl + US + CSCCT-FITC group, (II) PD + US + CSCC-FITC group, and (III) PD + US + CSCCT-FITC group, blue: nucleus; green: FITC-labeled nanoparticles; red: TH positive neurons (n = 3). The two-sided one-way ANOVA with a Tukey post-hoc analysis was used for comparison among multiple groups. *P < 0.05, **P < 0.01, ***P < 0.001, ****P < 0.0001, ns, not significant.

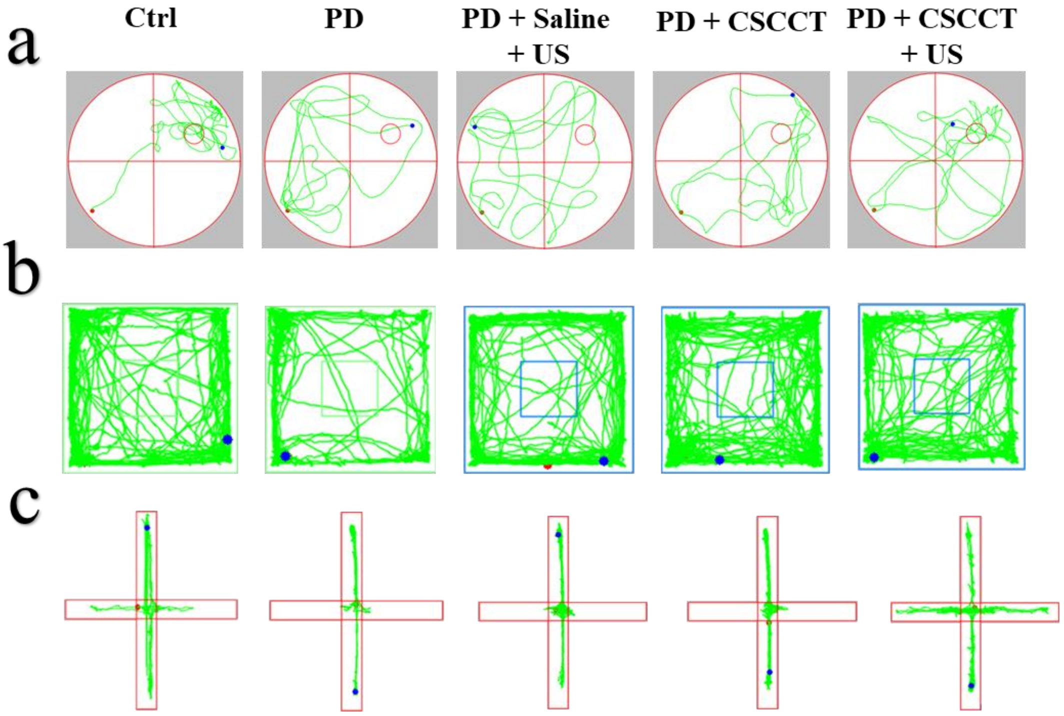

Figure S27. Representative paths of mice from different groups of mice obtained from a) the Morris water maze test, b) open field and c) elevated plus-maze from five different groups of mice (n = 8).

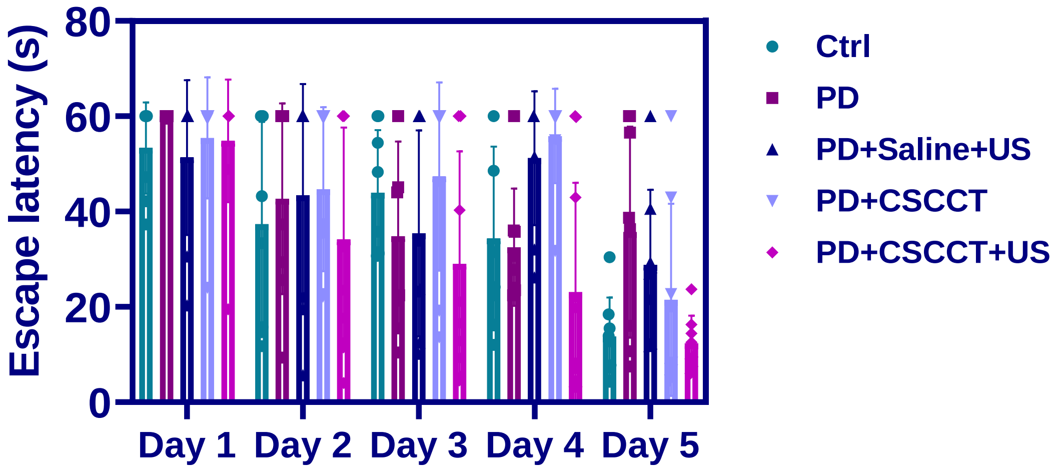

Figure S28. Histogram analysis of escape latency from five groups of mice obtained from the Morris water maze test (n = 8).

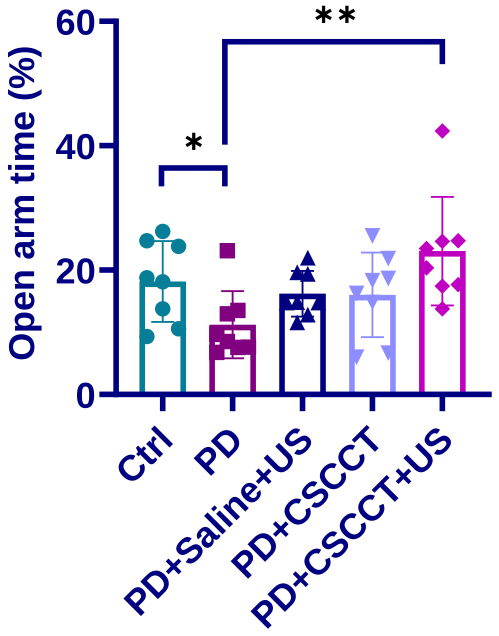

Figure S29. Percentage of open arm time in total time from five groups of mice in elevated plus-maze test (n = 8). The two-sided one-way ANOVA with a Tukey post-hoc analysis was used for comparison among multiple groups. *P < 0.05, **P < 0.01, ***P < 0.001, ****P < 0.0001, ns, not significant.

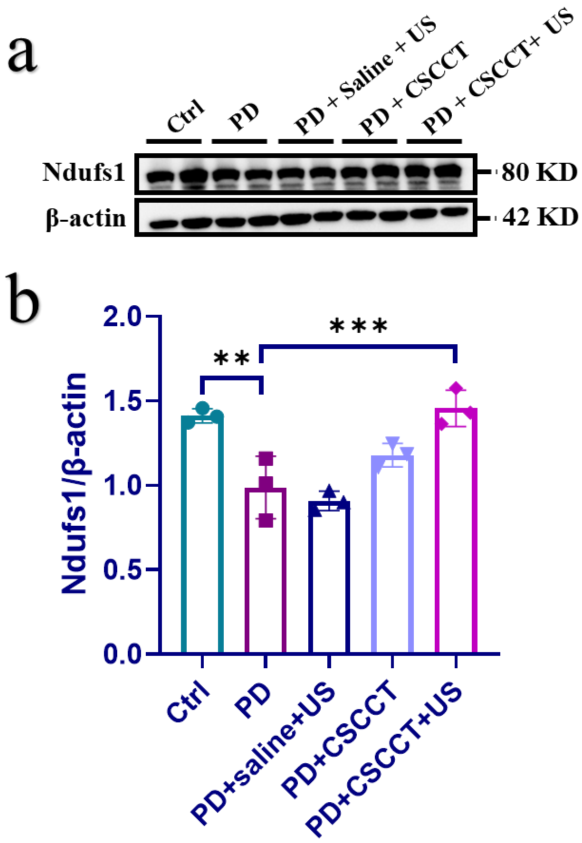

Figure S30. a) Western blot and b) relative quantitative analysis of Ndufs1 protein from different groups of mice (n = 3). The two-sided one-way ANOVA with a Tukey post-hoc analysis was used for comparison among multiple groups. *$P < 0.05$, **$P < 0.01$, ***$P < 0.001$, ****$P < 0.0001$, ns, not significant.

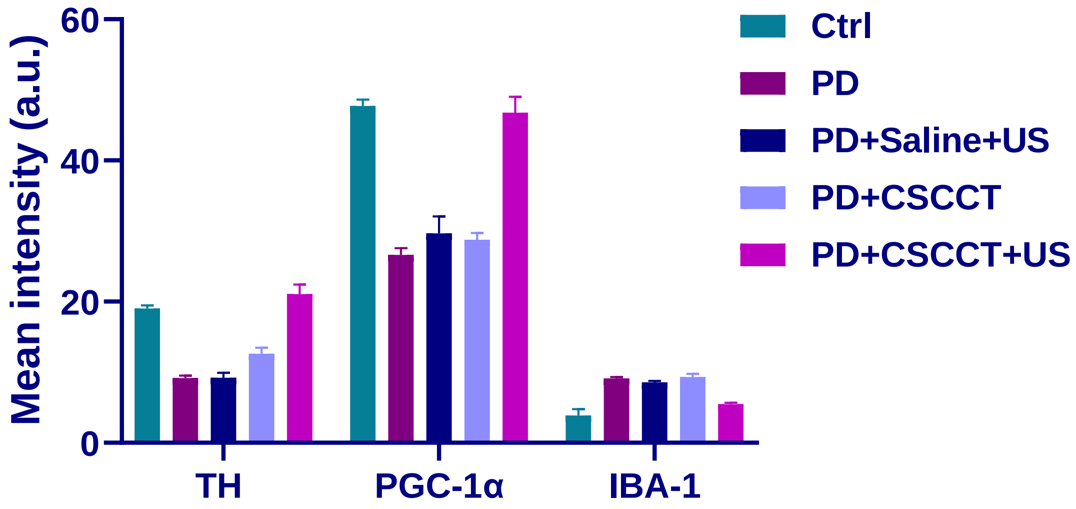

Figure S31. Quantitative analysis of fluorescence intensity from TH, PGC-1α and IBA-1in five groups of mice (n = 3).

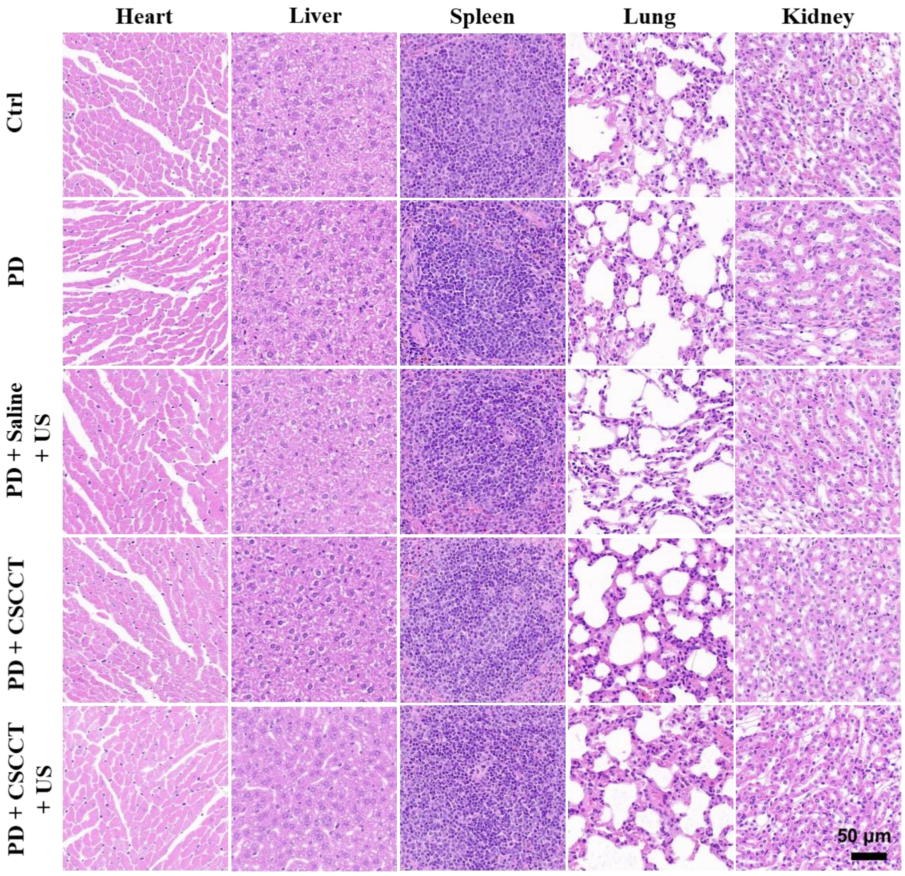

Figure S32. H&E staining images of major organs, including the heart, liver, spleen, lung and kidney collected from the different groups of mice (n = 3).
